# Looking at the full picture, using topic modeling to observe microbiome communities associated with disease

**DOI:** 10.1080/29933935.2024.2378067

**Published:** 2024-08-20

**Authors:** Rachel L. Fitzjerrells, Nicholas J. Ollberding, Ashutosh K. Mangalam

**Affiliations:** a Interdisciplinary Graduate Program in Informatics, University of Iowa, Iowa City, IA, USA; b College of Dentistry, University of Iowa, Iowa City, IA, USA; c Division of Biostatistics and Epidemiology; Cincinnati Children’s Hospital Medical Center; Department of Pediatrics, University of Cincinnati College of Medicine, Cincinnati, Ohio, USA; d Department of Pathology, Carver College of Medicine, University of Iowa, Iowa City, IA, USA; e Iowa City VA Health Care System, Iowa City, IA, USA

**Keywords:** Topic modeling, gut microbiome, bioinformatics, multiple sclerosis

## Abstract

The microbiome, a complex micro-ecosystem, helps the host with various vital physiological processes. Alterations of the microbiome (dysbiosis) have been linked with several diseases, and generally, differential abundance testing between the healthy and patient groups is performed to identify important bacteria. However, providing a singular species of bacteria to an individual as treatment has not been as successful as fecal microbiota transplant therapy, where the entire microbiome of a healthy individual is transferred. These observations suggest that a combination of bacteria might be crucial for the beneficial effects. Here we provide the framework to utilize topic modeling, an unsupervised machine learning approach, to identify a community of bacteria related to health or disease. Specifically, we used our previously published gut microbiome data of patients with multiple sclerosis (MS), a neurodegenerative disease linked to a dysbiotic gut microbiome. We identified communities of bacteria associated with MS, including genera previously discovered, but also others that would have been overlooked by differential abundance testing. This method can be a useful tool for analyzing the microbiome, and it should be considered along with the commonly utilized differential abundance tests to better understand the role of the gut microbiome in health and disease.

## Introduction

The microbiome is the collection of microorganisms that live in and on our body. Although, the microbiome includes bacteria, viruses, and fungi, the majority of microbiome studies have been focused on bacteria. With regard to the bacterial microbiome, it has been established that there is a community structure where a number of different species from various bacterial phyla live together. Their composition is regulated by various host and microbe-specific factors and in a steady state, they help to maintain homeostasis, keeping the host healthy. However, the alteration in the composition of the microbiome (dysbiosis) has been linked to a number of diseases including cancers, multiple sclerosis, Parkinson’s disease, Alzheimer’s disease, inflammatory bowel disease (IBD), and others.^1–4^

Recent articles outlining the best-practices for microbiome data analysis have highlighted several techniques that have allowed for microbial disease marker identification: including differential abundance comparisons of individual microbes (i.e., Wilcoxon rank-sum test), unsupervised clustering (i.e., PCoA), and supervised machine learning (i.e. Random Forest). However, the current methods lack a well-established unsupervised machine learning approach that can reveal a *community* of microbes linked to diseases or healthy state of the host.^5–7^ The microbiome is dependent on all organisms present; accordingly, it is important to study the community as a whole.

In detail, in the majority of microbiome studies, the relative abundance of each individual microbe is compared one at a time between people with a particular disease and healthy controls. This type of analysis has provided several major findings on overly enriched or overly depleted microbes that are linked to disease. For example, *Fusobacterium nucleatum* with colorectal cancer^8^ and *Clostridium difficile* with IBD.^9^ These findings are helpful in each respective area of research however, providing a singular species of bacteria to an individual lacking that species for health improvement has not been as successful as fecal matter transplant (FMT) therapy. With an FMT, where the entire microbiome is provided, the recipient can see improvement of disease.^10–12^ This reveals to us that the *community* of microorganisms is important to our health, and we should consider the structure of the community to better prevent, diagnose, and treat disease. FMTs do, however, have limited success possibly due to concerns that not all bacteria in the community may be responsible for the healthy phenotype. Therefore, there is a need for a method to identify microbiome communities linked to the healthy state of the host.

Thus, in this work, we aim to show the benefits of using the natural language processing (NLP) tool, topic modeling, in order to assess the community structure associated with health and/or diseases. Topic modeling is an unsupervised machine learning approach that assesses all the terms (bacteria) within documents (samples) and groups them into topics (communities) based on term similarities and patterns.

To do so, we utilized our previously published data on the gut microbiome composition of relapsing-remitting multiple sclerosis (RRMS) patients. RRMS is a neuroinflammatory autoimmune disease caused by genetic and environmental factors. The gut microbiome has emerged as a major environmental factor of interest in the development of RRMS as many studies have revealed that RRMS patients have a dysbiotic gut microbiome.^13–21^ As previous studies have focused on individual microbial differences, we instead applied topic modeling to our RRMS gut microbiome data to assess the latent interactions occurring among microbes and their association with RRMS. Specifically, we used the Latent Dirichlet Allocation (LDA) model as it allows documents to have fractional membership across topics.^22^ With topic modeling, we were able to confirm previously identified bacteria of interest linked with RRMS, but we additionally identified *communities* of bacteria, with otherwise overlooked bacteria, linked to RRMS. Therefore, we suggest topic modeling in addition to traditional approaches to better understand the microbiome of individuals with RRMS and other diseases with dysbiotic microbiome communities.

## Methods

### Clinical and sequence data

The clinical and 16s sequence data for our exploratory analysis were obtained from Chen et al. 2016,^14^ a prior publication from our group (RRMS = 31, HC = 36). The clinical and 16s sequence data for our validation analysis were obtained from Yadav et al. 2022,^3^ a separate publication from our group (RRMS = 20, HC = 33). For simplicity, each dataset is referred to using the first author’s last name (i.e., Chen and Yadav).

### 16s sequence data processing

Sequence data for the V3-V4 region of the bacterial 16s rRNA gene for each study were obtained from the National Center for Biotechnology Information (NCBI) Sequence Read Archive (SRA) under the BioProject numbers PRJNA335855 and PRJNA732670. The demographic data for all datasets can be found in Supplementary Table 1.

The sequence data was downloaded utilizing the SRA toolkit, denoised with DADA2^23^ using the default parameters and trimming of the forward and reverse reads at 240 and 180nt, respectively, for Chen, and trimming of the forward and reverse reads at 290 and 275nt, respectively, for Yadav. The taxonomy was assigned using the assignTaxonomy function and the Silva database (Version 138). Low prevalence features (relative abundance < 1e-5) were removed. Post-filtering, the reads were aggregated at the genus level for analysis.

### Statistical analysis and topic modeling

Statistical analyses were performed using the R environment for statistical computing and graphics (version 4.2.3). We first built a phyloseq^24^ object using the genus abundance table (i.e., genus-level phylotypes) and metadata to facilitate the statistical analysis. We filtered out genera with a prevalence less than 1e-5.

We utilized the FindTopicNumber function from the ldatuning^25^ package to identify an optimal latent topic number for our model based on the CaoJuan2009^26^ and Arun2010^27^ metrics. The method = “VEM” (variational expectation-maximization) option was selected to perform variational inference when deriving the latent topics. This was performed on each dataset and an average of the ideal topic numbers was selected. A total of 30 topics was chosen based on this approach (Supplementary Figure S1).

To derive the final set of topics, we used the LDA function from the topicmodels^28^ package to perform the latent Dirichlet allocation on each dataset. This model was chosen as it allows for fractional membership, or the allowance of assignment to multiple topics, when assigning reads to the underlying community types. We then extracted the beta and gamma probability matrices from our topic model using tidytext package^29^ and multiplied the per-document-per-topic probabilities by the read count for each sample to assign reads to each topic (i.e., generate a document-term matrix). A new phyloseq object was then built with the document-term matrix serving as the abundance table.

### Validation of topics found in original dataset

We extracted the topic-term-probability matrix from the Chen and Yadav LDA models and assessed the similarity in the community structure (topics) between our exploratory and validation data by calculating the cosine similarity matrix for each topic. We chose to utilize cosine similarity as it is often used to compare the similarity of two vectors in NLP analyses, including topic modeling.^30,31^ The communities that had a cosine similarity of 0.80 or higher were considered to reflect similar community types identified independently in each dataset, and thus validating the findings from the Chen data.

### Differential abundance of topics and bacteria within topics

To assess differences in the relative abundance of each community type between samples collected from RRMS patients and HC we performed a differential abundance analysis using the LinDA (linear models for differential abundance analysis) function from the MicrobiomeStat^32^ package with feature.dat.type = “count” and is.winsor = F. The Benjamini–Hochberg false discovery rate (FDR) correction was applied to account for the multiple testing. Community types with a p-value ≤0.05 and FDR ≤ 0.25 were considered significant.

To test differences in the genus-level relative abundance, we utilized our filtered phyloseq object, applied Total Sum Scaling (TSS) to 1e6 for normalization, and then performed the Wilcoxon Rank Sum Test. We chose this method as it is a typical approach in microbiome data analysis, and we wanted to present topic modeling alongside a widely used analysis method. The Benjamini–Hochberg false discovery rate (FDR) correction was applied to account for the multiple testing and a p-value ≤0.05 and FDR ≤ 0.25 were considered significant.

### Functional profiling of community topics

To improve our interpretation of the gut communities, we investigated the functional potential of the microbiome utilizing PICRUSt.^33^ The resulting pathway abundance table was filtered (relative abundance ≥ 1e-5) and normalized (TSS to 1e6). Data were analyzed in R with the Wilcoxon Rank Sum Test and FDR-adjusted with the Benjamini–Hochberg procedure. The same significance cutoffs were used (p-value ≤0.05 and FDR ≤ 0.25).

### Availability of data and materials

The sequence data used for analysis can be found at the National Center for Biotechnology Information (NCBI) Sequence Read Archive (SRA) under the BioProject numbers PRJNA335855 (*https://www.ncbi.nlm.nih.gov/bioproject/?term=PRJNA335855*) and PRJNA732670 (*https://ncbi.nlm.nih.gov/bioproject/?term=PRJNA732670*). On GitHub (*https://github.com/raeshrode/TheFullPicture_Article*) are the following data and scripts: (1) R script to analyze Chen data, Yadav data, and cosine similarity; (2) R environments after topic model analysis of Chen and Yadav datasets, and (3) abundance tables, metadata, and taxa tables for each dataset.

## Results

### Data for analysis

Our primary analysis was performed on the data from Chen et al. 2016^14^ and validated with the data from Yadav et al. 2022.^3^ For simplicity, each dataset is referred to by using the first author’s last name (e.g., Chen and Yadav). After data processing, we retained 175 genera in the Chen dataset and 160 in the Yadav validation dataset.

### Number and similarity of validated community types

**Figure 1.**
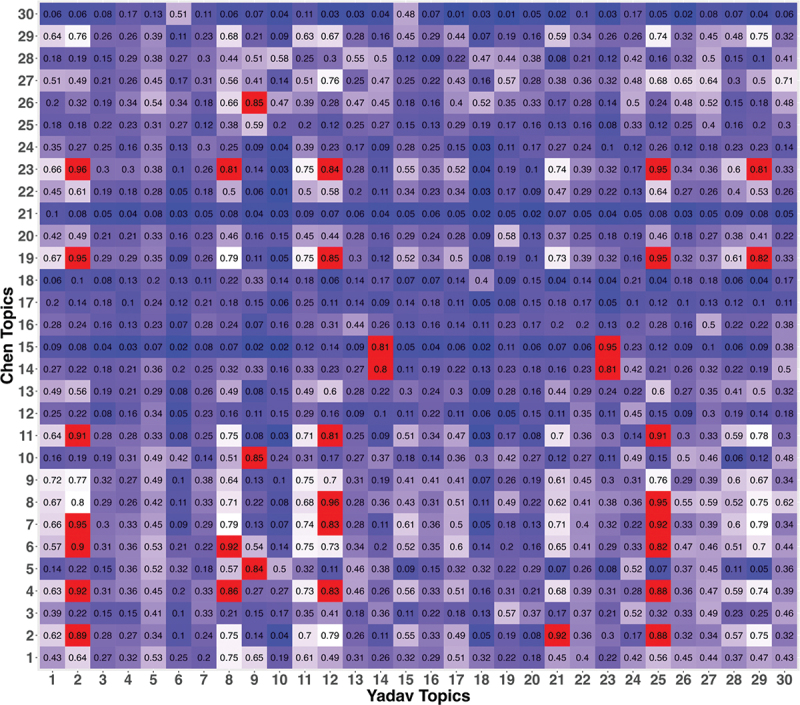
Validating microbiome community types in datasets. Community structure (topic) cosine similarity between Chen and Yadav datasets. A higher value reveals similar term assignment to topic. A value of 0.80 or greater was considered to reflect similar community types.

**Figure 2.**
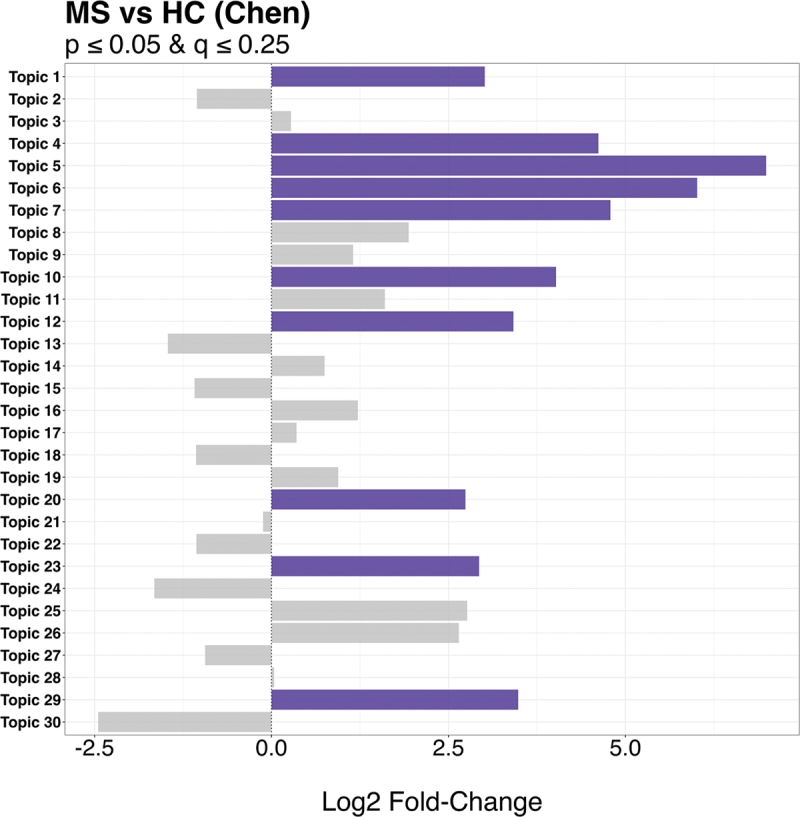
Identifying significant microbiome community types. Differentially abundant community types. Statistically significant topics assigned in Chen dataset highlighted in purple.

Our cosine similarity analysis revealed 34 community-type associations with high correlations (>0.80) between the Chen and Yadav topics (Figure 1), highlighting similarities in the community types across datasets. These associations comprised 13 topics from the exploratory dataset and nine from the validation dataset that were also associated with RRMS versus HC status. In the Chen dataset, 10 of the 30 topics were enriched in samples obtained from the RRMS patients compared to controls (Figure 2). In the Yadav dataset, four of the 30 topics were associated with RRMS versus HC status, with three enriched in RRMS and one enriched in HC samples. The plots for all statistically significant topic associations can be found in Supplementary Figure S2.

Specifically, of the significant community types found in the Chen data, five were validated based on having high cosine values (>0.80) to topics derived independently from the Yadav validation data. All these topics were significantly (*p* ≤ 0.05 and q ≤ 0.25) enriched in RRMS patients compared to HC. In detail, Chen Topic 4, Chen Topic 6, and Chen Topic 23 were similar to Yadav Topic 8 (cosine = 0.92, 0.86, 0.81, respectively). We will refer to this validated community as Community Type A (Figure 3A). Chen Topic 5 and Chen Topic 10 were similar to Yadav Topic 9 (cosine = 0.84, 0.85, respectively). We will refer to this validated community as Community Type B (Figure 3B).

**Figure 3.**
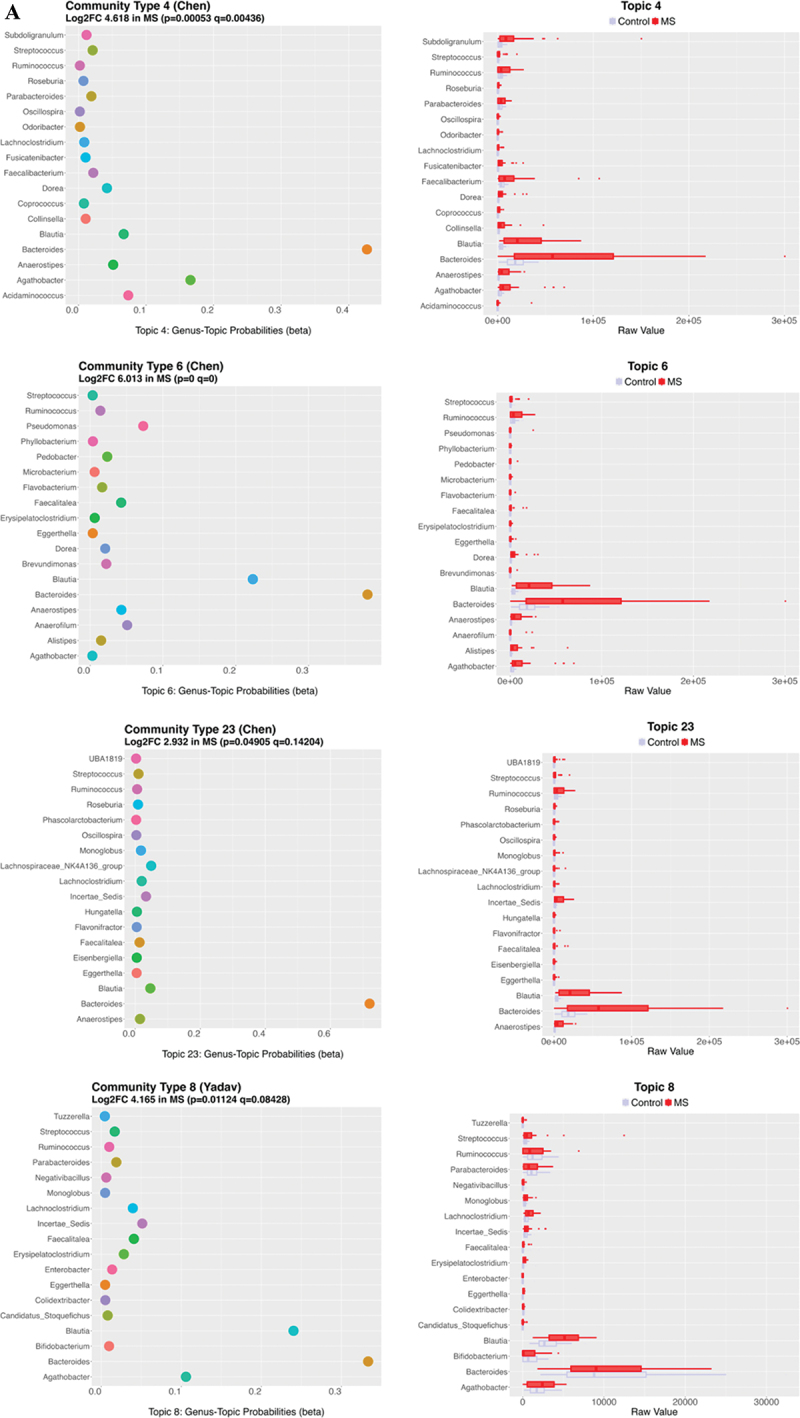
Community Type a in RRMS patients. MS gut microbiome Community Type a found in Chen and validated in Yadav. Specifically, communities Chen Topic 4, Chen Topic 6, Chen Topic 23, and Yadav Topic 8.

**Figure 3.**
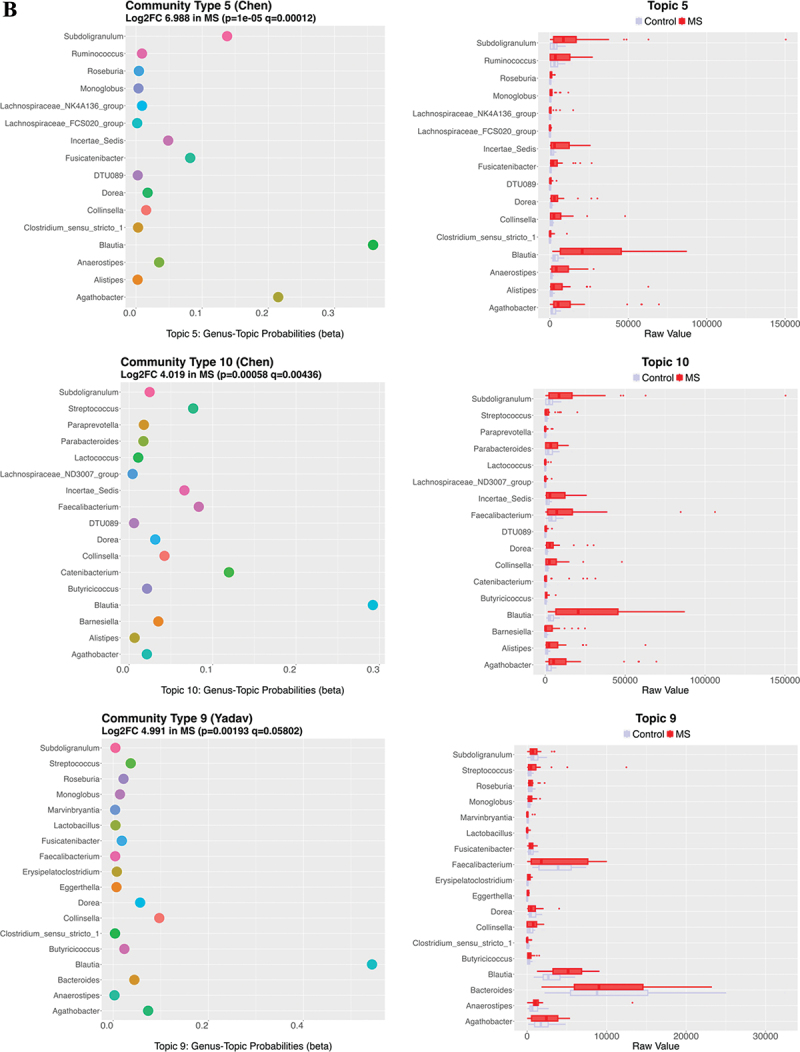
Community Type B in RRMS patients. RRMS gut microbiome Community Type B structures found in Chen and validated in Yadav. Specifically, communities Chen Topic 5, Chen Topic 10, and Yadav Topic 9.

### Highly assigned genera and their directional abundance in validated topics

We next examined the genera with high probabilities of assignment to these community types. In Community Type A, *Bacteroides* was the most often assigned genera (all topics), followed by *Blautia* (Chen 6, 23, Yadav 8), both were increased in RRMS compared to HC. Many other genera were also assigned to this community and were higher in RRMS than HC, including *Streptococcus* (all topics), *Eggerthella* (Chen 6, 23, Yadav 8), *Faecalitalea* (Chen 6, 23, Yadav 8), *Agathobacter* (Chen 6, Yadav 8) and *Lachnoclostridium* (Chen 4, 23, Yadav 8).

Several genera were assigned to Community Type A that varied in directional abundance between datasets. For example, *Ruminococcus* (all topics) was higher in the RRMS group in the Chen dataset and lower in the RRMS group in the Yadav dataset. Inversely, *Erysipelatoclostridium* (Chen 6, Yadav 8) was lower in RRMS in Chen and higher in RRMS in Yadav.

In Community Type B, *Blautia* (all topics) was the most often assigned genus and increased in RRMS compared to HC. Other genera enriched in RRMS and assigned to this community include *Dorea* (all topics), *Streptococcus* (Chen 10, Yadav 9), *Butryicoccus* (Chen 10, Yadav 9), *Roseburia* (Chen 5, Yadav 9), *Monoglobus* (Chen 5, Yadav 9), *Agathobacter* (all topics), and *Anaerostipes* (Chen 5, Yadav 9). Several other genera were important to Community Type B but varied in abundance between datasets. Specifically, *Subdoligranulum* (all topics), *Clostridium sensu stricto 1*(Chen 5, Yadav 9), and *Fusicatenibacter* (Chen 5, Yadav 9) were higher in RRMS patients in Chen, but lower in RRMS patients in Yadav.

### Differential abundance testing within topics

We assessed the assigned genera’s differential abundance within the statistically significant community types. Community Type A comprised many enriched genera; however, only *Blautia* was significantly increased in RRMS compared to HC in both the Chen (*p* = 2.37E–05, q = 0.001) and Yadav (*p* = 0.005, q = 0.121) datasets. None of the significantly depleted genera differed between RRMS and HC in abundance in both datasets. In Community Type B, the only shared significant finding between datasets was again the increase in *Blautia* in RRMS. All significantly altered bacteria are listed in Supplementary Tables 2 and 3.

### Functional potential of microbial communities

There were 152 significantly altered functional pathways identified in the Chen dataset and 72 in the Yadav dataset (Supplementary Tables 4 and 5). Of these pathways, 36 were shared between datasets with 15 increased in HC, and 21 increased in RRMS. Of note, METH-ACETATE-PWY was significantly enriched in RRMS patients.

## Discussion

We hypothesized that communities of microbes might be associated with RRMS patients when compared to healthy controls and that microbes not identified by one-at-a-time differential abundance testing approaches would be important to these dysbiotic community types. As such, we utilized our previously published data and performed topic modeling on this dataset to look for community types associated with RRMS. Out of 30 topics assessed, we identified 10 that were more often associated with RRMS when compared to HC, and we validated these findings utilizing a separate dataset.

Several themes were found in Community Type A and Community Type B, suggesting similar dysbiotic communities associated with RRMS. We found that *Bacteroides* was one of the most often assigned genera. This genus was higher in RRMS than HC in Chen, and validated in Yadav, but did not reach statistical significance at this sample size in either dataset. This finding highlights the possibility that differences in clusters of microbes might be more important than differences in specific microbes in the dysbiotic gut microbiome communities seen in MS patients. *Blautia* also had a high assignment probability and was enriched in RRMS patients in both datasets. In multiple studies, *Blautia* has been linked to MS.^20,34^ Functionally its enrichment and depletion in the gut have both been linked to inflammatory diseases (enrichment: breast cancer,^4^ inflammatory bowel syndrome,^35^ and MS;^13,34^ depletion: Crohn’s disease,^36^ colorectal cancer,^1^ and MS^20^). Additionally, *Dorea* was highly assigned to these Community Types, and although *Dorea* is usually considered a gut commensal, its increased abundance has been linked to MS^34^ and other inflammatory diseases such as Crohn’s disease.^35^ Specifically, the pro-inflammatory effects of *Dorea* could be due to the ability of some species of *Dorea* to induce IFNy, metabolize sialic acids, and degrade mucin.^37,38^ Furthermore, *Blautia* utilizes gases produced by *Dorea*,^39^ thus these inter-bacterial interactions could be important to the gut microbiome community impact on RRMS patients.

*Eggerthella, Roseburia*, and *Anaerostipes*, were also assigned to these RRMS community topics and found to be higher in RRMS compared to HC. *Eggerthella* was identified in the Yadav dataset as being significantly higher. This increased abundance has been identified in multiple MS studies^15,19,34^ and other autoimmune disorders like systemic lupus erythematosus.^40^ Although we found *Anaerostipes* and *Roseburia* to be increased in RRMS, other studies have found the inverse.^21,34^ Even with these differences, this highlights that these genera are important to the RRMS community structure and future studies are needed to elicit their function and apparent abundance changes.

Several genera linked to gut permeability were assigned to the RRMS community types including *Streptococcus, Lachnoclostridium, Faecalibacterium*, and *Faecalitalea*. *Streptococcus* had a higher relative abundance in RRMS patients compared to HC, but again associations for this specific genus did not reach statistical significance. *Streptococcus* species have been shown to cross the epithelial barrier and translocate systemically,^41^ thus have the ability to induce systemic inflammation. They can also cross the blood-brain-barrier (BBB),^42,43^ which is of interest in MS research, as the gut-brain axis is often implicated in the pathobiology of this disease. One hypothesized mechanism of action is that inflammation of the intestinal barrier, potentially due to a lack of short-chain fatty acids (SCFAs) or other immunological changes, results in gut dysbiosis.^44^ This allows pathogens and bacterial products to either affect the CNS directly through neuro-immune-endocrine pathways or indirectly by inducing systemic inflammation due to the translocation of bacteria and their products into the bloodstream and then to the CNS. As several microbes and microbial by-products have been identified in the CNS of MS patients,^45^ the gut-brain axis has gained traction and is important to consider when understanding the etiopathology of MS. Additionally, higher abundances of *Lachnoclostridium* have been linked to reduced levels of acetate^46^ and through our functional analysis we saw METH-ACETATE-PWY was increased in RRMS patients in both datasets. This is the “Methanogenesis from Acetate Pathway”, a pathway that reduces acetate. Acetate is an SCFA that has been associated with a healthy gut microbiome and a developed immune system;^47^ reduced levels might result in a compromised gut as well as blood–brain barrier in RRMS patients. To note, however, gut dysbiosis is influenced bi-directionally in the gut-brain-axis, and the CNS can have direct and indirect effects on the gut microbiome (i.e., endocrine mediators and catecholamines concentrations).^48^

*Faecalibacterium* was also assigned to these community types and was lower in RRMS patients than HC, in fact identified as significantly lower in the Chen dataset. This genus is a butyrate-producer and linked to a decrease in intestinal inflammation.^49^ Thus, along with the increase of several genera, a decrease in others such as *Faecalibacterium* are important to the community structure of the dysbiotic gut microbiome of RRMS patients and possibly gut permeability. Of note, *Faecalitalea* was assigned to the RRMS community types and was increased in RRMS patients compared to HC. This genus is thought to be beneficial as it can ferment many sugars and its major end product is also butyric acid.^50^ Butyric acid is considered to support the integrity of the gut.^51^

As for differences found between our exploratory and validation data, the abundance of several bacteria varied between Chen and Yadav. For example, in Community Type A, *Ruminococcus* was higher in the Chen RRMS group but lower in the Yadav RRMS group compared to their corresponding HCs. And the inverse was found for *Erysipelatoclostridium*. As for Community Type B, three bacteria were higher in Chen RRMS group but lower in Yadav RRMS group: *Subdoligrandulum, Clostridium sensu strictro 1*, and *Fusicantenibacter*. Although the abundances varied between datasets, the presence of the microbes within the topics could be more influential to the community dynamic than the relative abundance.

Collectively, our findings indicate that the complex dysbiotic microbiota in RRMS patients can be characterized by a diverse community of bacteria specifically comprising a reduction in beneficial symbiont bacteria, an increase in potentially harmful pathogenic bacteria, and an overall shift of certain commensal bacteria toward a pathobiont phenotype. As a number of bacteria in these communities don’t reach statistical significance on their own, our findings highlight that the collective impact of these bacteria is greater than their individual effect. Thus, a healthy or disease phenotype outcome can be attributed to the balance between symbionts and pathobionts shifting toward pathobionts. It seems there are certain keystone symbionts species, such as *Faecalibacterium*, which are mostly associated with a healthy phenotype, likely due to their inability to adjust to environmental changes lacking nutritional sources such as dietary fibers.^52^ However, other commensal gut bacteria, such as *Bacteroides*, *Blautia*, and *Eggerthella spp.*, can be more flexible due to their adaptability to thrive in diverse conditions and utilize a wide range of substrates as a food source. They can efficiently switch their metabolic pathways and enzymatic activities to utilize different nutrients, ensuring their survival and maintenance in the ever-changing gut ecosystem.^53,54^

However, there are several unknowns, such as what are the factors promoting dysbiosis, why certain individuals are more prone than others, and most importantly, whether dysbiosis can be corrected through diet (prebiotic) or microbiota replacement (probiotic) or both (symbiotic). In detail, to disentangle the complex interplay between the microbiome and its host, longitudinal studies with large-scale population cohorts are crucial. Analyzing the microbiome before and after disease onset in these studies will provide valuable insights into how the microbiome impacts the host and how, in turn, the host influences the microbiome. This understanding would help harness the enormous potential of the gut microbiome as a future diagnostic and therapeutic agent.

Our findings here are in line with many prior findings on the dysbiotic gut microbiome of RRMS patients. In addition, with the use of topic modeling, we observed associations for community structures related to RRMS that cannot be identified with differential abundance testing, including patterns of inter-bacterial interactions (i.e., *Blautia* and *Dorea* in Community Type B). These findings should be further validated with more datasets, other sequencing methods such as shotgun sequencing, and diverse cohorts but highlight the potential of topic modeling in microbiome research. In the future, we hope that topic modeling will be incorporated with traditional statistical approaches for microbiome analysis and help provide a better picture of the microbiome as a whole in complex diseases such as RRMS.

## Supplementary Material

corrections_supplementary_data cleaned.docx
