# Supplementary material for: Looking at the full picture, using topic modeling to observe microbiome communities associated with disease
Source: Gut Microbes Rep. 2024 Aug 20;1(1):2378067. doi: 10.1080/29933935.2024.2378067 (PMC11340690; doi:10.1080/29933935.2024.2378067)
Supplement: corrections_supplementary_data cleaned.docx [file KGMR_A_2378067_SM3295.docx]

**Supplementary Figures**

**Supplementary Figure 1**: The ideal topic number for each dataset is at the minimum value that both metrics generally reach. For the Chen dataset 33 topics was ideal and for Yadav 27 topics was ideal. On average, 30 topics is ideal for these datasets and was selected.

a)


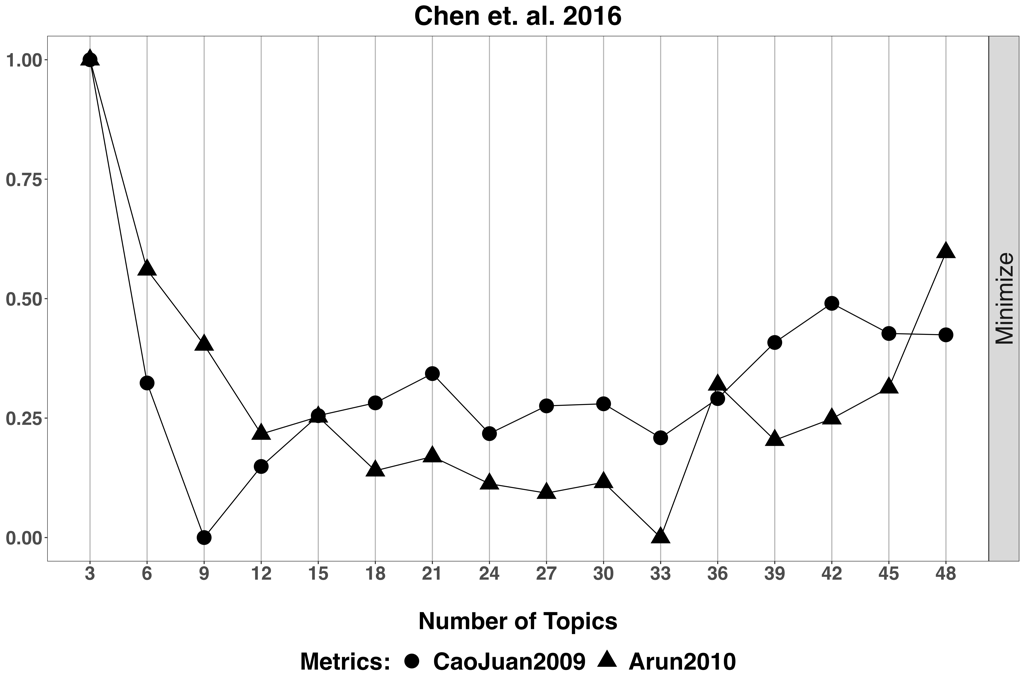


b)


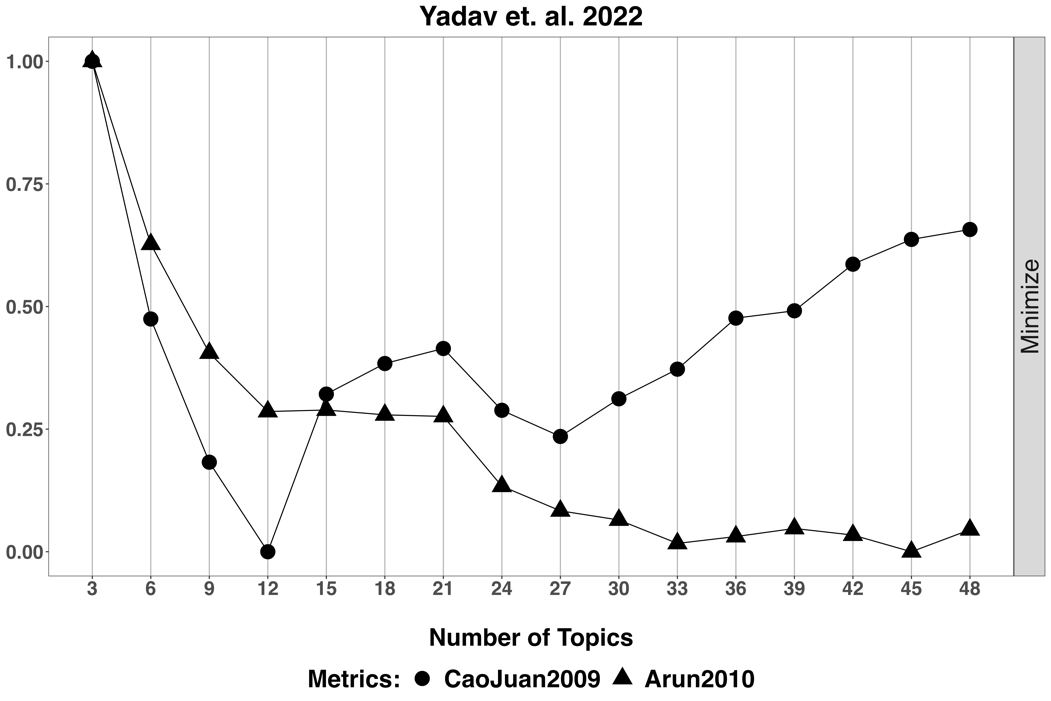


**Supplementary Figure 2**: a) The significant topics found in the Chen dataset not shown in the results. b) The significant topics found in the Yadav dataset not shown in the results. The left-hand side contains the probability of the genus being assigned to the topic. The right-hand side contains the abundance comparison between MS and HC of the highly assigned bacteria. Boxplots fill is based on group assignment.


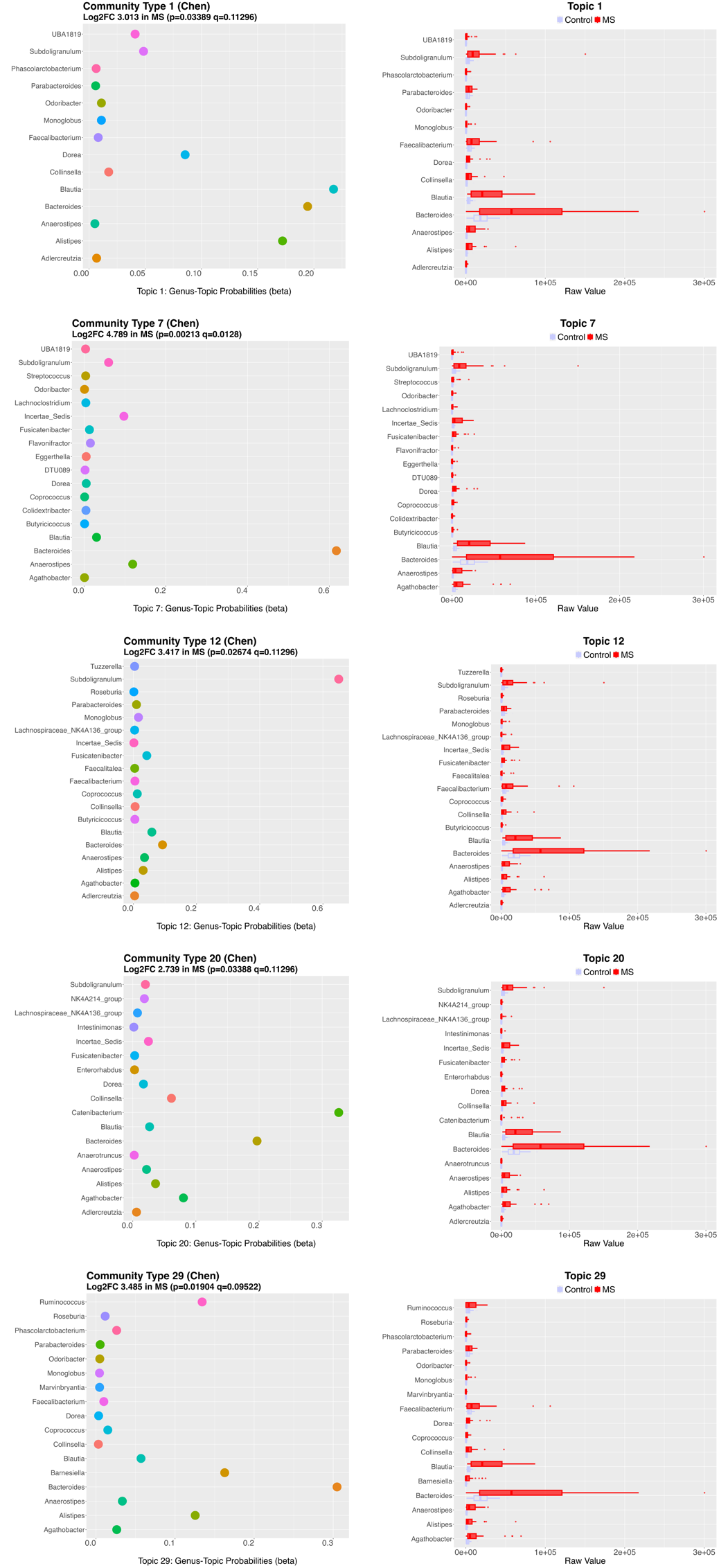


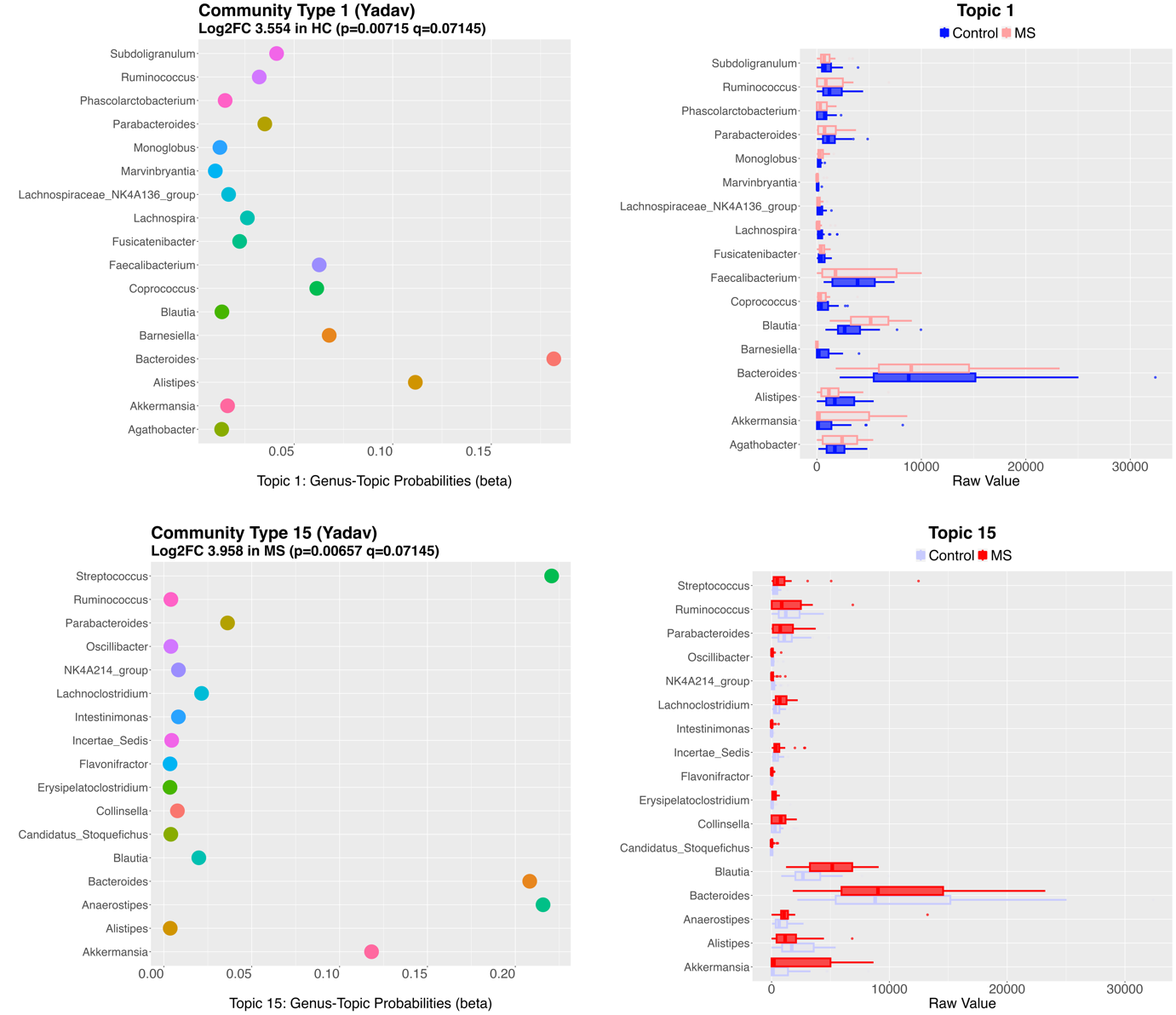


**Supplementary Table 1**: Demographics for all Datasets

|  | **RRMS** (Chen) | **RRMS** (Yadav) | **HC** (Chen) | **HC** (Yadav) |
| --- | --- | --- | --- | --- |
| **N** | 31 | 20 | 36 | 33 |
| **Sex (M/F)** | 10/21 | 5/15 | 14/22 | 5/28 |
| **Age (SD)** | 39.3 (10.6) | 43 (7.7) | 45.2 (10.2) | 42 (14) |
| **BMI (SD)** | 32.7 (7.4) | 30 (7.9) | 28.0 (6.3) | 24 (3.7) |

RRMS: relapsing-remitting multiple sclerosis

HC: healthy controls

SD: Standard Deviation

**Supplementary Table 2**: Bacteria in Chen Dataset

| **Chen Genera** | **p** | **q** | **MS mean** | **MS SD** | **HC mean** | **HC SD** |
| --- | --- | --- | --- | --- | --- | --- |
| Actinomyces | 0.270330509 | 0.61977795 | 296.0551872 | 789.5544739 | 134.4532252 | 227.1449162 |
| Bifidobacterium | 0.776321651 | 0.906783602 | 126.7713299 | 351.3350195 | 273.5793901 | 1100.918413 |
| Rhodococcus | 0.056289014 | 0.307830545 | 47.08037071 | 245.1835885 | 0 | 0 |
| Microbacterium | 0.00111524 | 0.024395868 | 207.1704439 | 1110.641293 | 0 | 0 |
| Cutibacterium | 0.056289014 | 0.307830545 | 53.41785073 | 278.2611597 | 0 | 0 |
| Propionibacterium | 0.733410709 | 0.906783602 | 292.5670742 | 1543.292562 | 53.56431821 | 172.0923308 |
| Libanicoccus | 0.232931449 | 0.617593186 | 12.50182954 | 68.47534047 | 371.8186658 | 1992.858152 |
| Olsenella | 0.464531184 | 0.759747263 | 24.9439324 | 135.240901 | 4.652003551 | 27.91202131 |
| Collinsella | 0.254001053 | 0.617593186 | 24014.57325 | 26734.42435 | 17347.73511 | 19569.71519 |
| Raoultibacter | 0.864022265 | 0.939154636 | 10.08603287 | 50.22222381 | 4.741505526 | 20.07738611 |
| Adlercreutzia | 0.693379219 | 0.906783602 | 2025.260844 | 1905.773111 | 1816.519592 | 1901.64818 |
| DNF00809 | 0.220841625 | 0.617593186 | 191.5583749 | 233.7074093 | 216.4541785 | 416.4040842 |
| Eggerthella | 0.065460976 | 0.327913101 | 2775.200744 | 4757.206742 | 1244.290404 | 1919.308985 |
| Enterorhabdus | 0.16508756 | 0.506847771 | 214.6764878 | 940.3003628 | 437.8189122 | 1723.573277 |
| Gordonibacter | 0.943048456 | 0.959496975 | 597.352 | 1125.185864 | 435.3912481 | 484.9812486 |
| Senegalimassilia | 0.611956376 | 0.836659108 | 136.4108219 | 356.3669453 | 309.8393135 | 1103.332404 |
| Slackia | 0.328818208 | 0.640032563 | 459.5026995 | 1010.919227 | 1039.861125 | 2021.460551 |
| Bacteroides | 0.509728443 | 0.783818191 | 263696.3133 | 167810.5789 | 283335.6816 | 159402.9114 |
| Barnesiella | 0.132442352 | 0.463548232 | 15444.58847 | 25240.88998 | 8815.805624 | 14037.63074 |
| Coprobacter | 0.899204624 | 0.959496975 | 295.1385298 | 588.9469575 | 325.4470186 | 880.1972695 |
| Dysgonomonas | 0.377537887 | 0.667364951 | 0 | 0 | 111.859412 | 671.156472 |
| Butyricimonas | 0.941253768 | 0.959496975 | 1738.294165 | 3180.65364 | 1704.774651 | 2810.515772 |
| Odoribacter | 0.510601565 | 0.783818191 | 2302.936638 | 3190.085416 | 2241.393456 | 3113.057707 |
| Sanguibacteroides | 0.025991986 | 0.189524898 | 82.44483276 | 293.9482827 | 0 | 0 |
| Muribaculum | 0.123710756 | 0.451070795 | 26.18564558 | 121.7646904 | 0 | 0 |
| Alloprevotella | 0.717636922 | 0.906783602 | 232.5359683 | 648.0682536 | 1690.003218 | 5700.378162 |
| Paraprevotella | 0.958280746 | 0.969359136 | 660.6695703 | 1838.509061 | 3900.875842 | 9514.387798 |
| Prevotella | 0.241592988 | 0.617593186 | 10467.2109 | 46284.5362 | 45399.55443 | 106960.9547 |
| Prevotellaceae_NK3B31_group | 0.527710819 | 0.786891007 | 144.4461699 | 667.5661154 | 111.5542077 | 589.1726597 |
| Prevotellaceae_UCG-001 | 0.295663246 | 0.621516019 | 1058.974073 | 4622.36403 | 333.8612948 | 1890.195886 |
| Prevotellaceae_UCG-003 | 0.930889454 | 0.959496975 | 37.6071295 | 205.9827315 | 373.489428 | 2240.936568 |
| Prevotellaceae_UCG-004 | 0.123710756 | 0.451070795 | 269.8708439 | 1476.911245 | 0 | 0 |
| Alistipes | 0.235313762 | 0.617593186 | 25586.82772 | 29283.19939 | 19120.18619 | 28513.199 |
| Rikenellaceae_RC9_gut_group | 0.000393135 | 0.011466437 | 1601.428925 | 4034.656167 | 4071.572423 | 13722.65785 |
| Parabacteroides | 0.144661007 | 0.495697415 | 20250.62018 | 29462.91907 | 40831.27156 | 51731.82143 |
| Flavobacterium | 0.000328283 | 0.011466437 | 2266.262209 | 9696.257358 | 10.13869447 | 23.40079841 |
| Pedobacter | 3.43E-07 | 3.00E-05 | 921.7115125 | 4231.48098 | 2.246484252 | 13.47890551 |
| Bacillus | 0.249285069 | 0.617593186 | 24.14077877 | 126.6243322 | 29.3790694 | 176.2744164 |
| Solibacillus | 0.377537887 | 0.667364951 | 0 | 0 | 32.55518501 | 195.3311101 |
| Candidatus_Stoquefichus | 0.528023802 | 0.786891007 | 318.6397443 | 1197.302238 | 266.7442975 | 1123.269828 |
| Catenibacterium | 0.006390732 | 0.101670738 | 8828.50822 | 18569.99608 | 11963.51262 | 40851.05294 |
| Coprobacillus | 0.634603266 | 0.851013401 | 48.67576076 | 166.5260643 | 172.2634214 | 731.8782031 |
| Erysipelatoclostridium | 0.158261438 | 0.504761298 | 941.2419692 | 1415.346484 | 1494.839456 | 1908.719136 |
| Erysipelotrichaceae_UCG-003 | 0.089970303 | 0.403712898 | 8393.376589 | 14874.89162 | 14659.68585 | 21796.15186 |
| Catenisphaera | 0.112357302 | 0.451070795 | 0 | 0 | 4299.472128 | 25772.64138 |
| Dielma | 0.634588214 | 0.851013401 | 60.47511326 | 133.9878755 | 134.6274158 | 378.2267286 |
| Faecalitalea | 0.025100487 | 0.189524898 | 5272.805523 | 11662.41388 | 1793.158385 | 4508.967992 |
| Holdemanella | 0.383847033 | 0.671732307 | 3781.46165 | 8476.402214 | 3875.567318 | 10775.82674 |
| Holdemania | 0.390974371 | 0.67743084 | 434.0467809 | 703.3517862 | 370.6651034 | 455.8030914 |
| Merdibacter | 0.113479328 | 0.451070795 | 177.2823744 | 446.998363 | 377.5726509 | 888.7696991 |
| Turicibacter | 0.181572853 | 0.538563547 | 558.2362368 | 935.6045159 | 2102.401183 | 4488.849752 |
| Granulicatella | 0.914429673 | 0.959496975 | 17.59993644 | 34.79917743 | 31.13380465 | 76.83219638 |
| Enterococcus | 0.05901235 | 0.312944279 | 73.15768287 | 286.2078011 | 597.7517911 | 1656.23765 |
| Lactobacillus | 0.008019346 | 0.110576244 | 855.4469433 | 2911.72023 | 6380.650887 | 16993.66538 |
| Lactococcus | 0.354346517 | 0.659687665 | 558.3483249 | 1108.375997 | 619.4208161 | 1613.589528 |
| Streptococcus | 0.333238919 | 0.640844076 | 6933.629776 | 8584.386916 | 12909.1928 | 19490.08241 |
| Gemella | 0.0088461 | 0.110576244 | 27.42108364 | 53.20768893 | 14.47234191 | 51.68916437 |
| Christensenella | 0.455017762 | 0.758362936 | 294.6782524 | 571.0217954 | 160.7148154 | 255.8176073 |
| Christensenellaceae_R-7_group | 0.323943168 | 0.640032563 | 18155.25007 | 26129.39464 | 24868.51601 | 27698.33127 |
| Ruminiclostridium | 0.329159604 | 0.640032563 | 21.60435641 | 85.48271697 | 132.2371037 | 487.0393277 |
| Clostridium_sensu_stricto_1 | 0.766695114 | 0.906783602 | 2608.164379 | 6187.373209 | 2696.031488 | 5496.518654 |
| Anaerofustis | 0.589254425 | 0.818408924 | 215.037688 | 267.4999395 | 160.680861 | 202.3920625 |
| Eubacterium | 0.147292946 | 0.495697415 | 92.15180051 | 231.6533215 | 32.85538825 | 131.8087802 |
| Defluviitaleaceae_UCG-011 | 0.528768706 | 0.786891007 | 256.3836005 | 603.4009177 | 289.3502712 | 367.6339357 |
| A2 | 0.286868651 | 0.61977795 | 121.1092281 | 663.3425614 | 0 | 0 |
| Agathobacter | 0.235313762 | 0.617593186 | 51165.86495 | 46949.80372 | 36435.78143 | 38123.91054 |
| Anaerostipes | 0.005427922 | 0.096356085 | 31563.43873 | 23914.90732 | 18179.33089 | 17787.01464 |
| Blautia | 2.37E-05 | 0.001035916 | 122708.3764 | 88253.1636 | 58345.07743 | 45593.9949 |
| CAG-56 | 0.261333998 | 0.618019591 | 92.01967156 | 283.791987 | 4.709412802 | 22.63174566 |
| Coprococcus | 0.423237557 | 0.726142868 | 7808.468122 | 7371.497384 | 5982.639049 | 7196.373826 |
| Dorea | 0.096662792 | 0.412585087 | 19735.56781 | 19207.69216 | 12533.49249 | 11498.51591 |
| Eisenbergiella | 0.973495341 | 0.973756783 | 1013.573682 | 1919.14195 | 607.5151127 | 791.671163 |
| Epulopiscium | 0.005506062 | 0.096356085 | 32.44645591 | 97.65812947 | 0 | 0 |
| Frisingicoccus | 0.504297561 | 0.783818191 | 141.8618007 | 279.346177 | 184.360433 | 402.0732398 |
| Fusicatenibacter | 0.066481732 | 0.327913101 | 16673.86086 | 18742.76979 | 8770.308958 | 9851.595192 |
| GCA-900066575 | 0.198043664 | 0.577627354 | 142.1045055 | 198.2582849 | 110.6067349 | 187.0844154 |
| GCA-900066755 | 0.008533615 | 0.110576244 | 20.42148804 | 47.87477795 | 6.649805809 | 25.5671273 |
| Howardella | 0.076575524 | 0.362181532 | 13.61987933 | 74.59915141 | 184.5768036 | 511.4447751 |
| Hungatella | 0.03799087 | 0.246237123 | 346.8458509 | 1120.364102 | 113.3079263 | 416.7497032 |
| Lachnoclostridium | 0.13022797 | 0.463548232 | 3809.820667 | 4471.090847 | 2694.348956 | 4252.144158 |
| Lachnospira | 0.540591608 | 0.788362761 | 1039.913976 | 1160.443168 | 1070.583111 | 1129.008367 |
| Lachnospiraceae_AC2044_group | 0.73035958 | 0.906783602 | 66.10099549 | 231.5262726 | 45.44454068 | 119.305926 |
| Lachnospiraceae_FCS020_group | 0.532311049 | 0.786891007 | 422.6686139 | 545.9112154 | 521.7417813 | 591.9416509 |
| Lachnospiraceae_ND3007_group | 0.777243087 | 0.906783602 | 751.8188584 | 1838.151296 | 623.9869469 | 1142.01614 |
| Lachnospiraceae_NK4A136_group | 0.742049649 | 0.906783602 | 3853.177003 | 8076.018904 | 3796.708209 | 6018.714305 |
| Lachnospiraceae_NK4B4_group | 0.837153085 | 0.93214418 | 383.1971441 | 1114.170628 | 90.78833502 | 198.037832 |
| Lachnospiraceae_UCG-001 | 0.375232727 | 0.667364951 | 289.7814514 | 561.0568495 | 279.1401684 | 552.6976762 |
| Lachnospiraceae_UCG-004 | 0.158639265 | 0.504761298 | 128.0555192 | 414.7919615 | 40.34974872 | 88.37741436 |
| Lachnospiraceae_UCG-010 | 0.698755648 | 0.906783602 | 465.7151133 | 402.1398903 | 620.5457423 | 812.8133907 |
| Lachnospiraceae_XPB1014_group | 0.025991986 | 0.189524898 | 12.1552832 | 45.16962423 | 0 | 0 |
| Lactonifactor | 0.012086125 | 0.124415988 | 192.1203153 | 961.4941282 | 8.129549936 | 35.92729703 |
| Marvinbryantia | 0.76886624 | 0.906783602 | 1029.505365 | 2140.53589 | 791.8139088 | 983.7906112 |
| Moryella | 0.535085885 | 0.786891007 | 62.21887018 | 101.4429611 | 47.21269513 | 84.67026286 |
| Roseburia | 0.771998033 | 0.906783602 | 4770.4695 | 5782.326158 | 7069.59532 | 11406.42686 |
| Sellimonas | 0.924315124 | 0.959496975 | 276.5940758 | 801.8210407 | 222.7024577 | 526.3811759 |
| Shuttleworthia | 0.506052166 | 0.783818191 | 92.99895143 | 159.5663487 | 197.3885496 | 548.1823112 |
| Tuzzerella | 0.298327689 | 0.621516019 | 591.771065 | 1237.458329 | 1617.543625 | 4980.630855 |
| Tyzzerella | 0.16508756 | 0.506847771 | 117.2302885 | 317.8708486 | 435.2509845 | 1843.984011 |
| UC5-1-2E3 | 0.745497409 | 0.906783602 | 99.14589122 | 272.9883493 | 54.81550136 | 98.59049009 |
| Monoglobus | 0.56034771 | 0.80073217 | 7259.605304 | 8275.3618 | 5111.048917 | 4682.750579 |
| Butyricicoccus | 0.230256817 | 0.617593186 | 2925.7608 | 2272.466977 | 5637.511898 | 6237.614256 |
| UCG-008 | 0.857034594 | 0.939154636 | 18.70961244 | 49.86802293 | 371.7181307 | 2056.896599 |
| UCG-009 | 0.73143709 | 0.906783602 | 79.05885073 | 178.5330823 | 115.1072664 | 205.4276107 |
| Acetanaerobacterium | 0.123722275 | 0.451070795 | 22.39123976 | 49.58993547 | 53.43829904 | 70.96312344 |
| Ethanoligenens | 0.313023089 | 0.636965587 | 203.2494407 | 816.9682751 | 489.6244453 | 1682.788321 |
| Colidextribacter | 0.353646343 | 0.659687665 | 1675.116792 | 2057.75323 | 2093.203604 | 1912.392943 |
| Flavonifractor | 0.000794926 | 0.019873157 | 3816.009611 | 8358.146667 | 678.938268 | 931.3978514 |
| Intestinimonas | 0.436148667 | 0.733904006 | 1168.357478 | 3341.44401 | 1018.869709 | 1965.564908 |
| NK4A214_group | 0.483733639 | 0.783818191 | 2466.764578 | 3600.501992 | 5685.78282 | 8167.311289 |
| Oscillibacter | 0.460568743 | 0.759747263 | 434.5781615 | 1510.3025 | 763.9915152 | 2553.326256 |
| Oscillospira | 0.051805158 | 0.307830545 | 1620.002495 | 1815.885518 | 957.1853333 | 1138.485604 |
| Pseudoflavonifractor | 0.09452484 | 0.412585087 | 48.57497287 | 135.2700944 | 8.773850364 | 32.72956066 |
| UCG-002 | 0.732812883 | 0.906783602 | 14881.70741 | 19572.64046 | 12413.32723 | 12650.2269 |
| UCG-003 | 0.179960081 | 0.538563547 | 2423.265979 | 6209.054878 | 897.4286256 | 1708.05189 |
| UCG-005 | 0.431339507 | 0.732858386 | 3119.953745 | 7305.800078 | 4097.103428 | 5944.640386 |
| UCG-007 | 0.821833146 | 0.927876132 | 34.64776196 | 90.41950086 | 28.45822029 | 63.07697884 |
| Hydrogenoanaerobacterium | 0.597687628 | 0.823585314 | 227.8500515 | 1027.444885 | 198.2377831 | 916.616195 |
| Anaerofilum | 0.254095482 | 0.617593186 | 5137.252866 | 17114.46479 | 1820.860217 | 6179.425026 |
| Anaerotruncus | 0.637044317 | 0.851013401 | 913.9641638 | 2109.527679 | 736.9699349 | 1039.984567 |
| Angelakisella | 0.756842991 | 0.906783602 | 36.66047414 | 129.0081098 | 19.60491986 | 59.8886544 |
| CAG-352 | 0.211826597 | 0.607699253 | 176.1986058 | 386.9198846 | 1123.64825 | 4882.860049 |
| Candidatus_Soleaferrea | 0.841593031 | 0.93214418 | 269.8199314 | 342.3629277 | 256.904583 | 308.6396914 |
| Caproiciproducens | 0.243732853 | 0.617593186 | 70.49606382 | 131.7948362 | 105.4632174 | 145.1706398 |
| DTU089 | 0.259406146 | 0.618019591 | 2185.302892 | 2493.924795 | 1657.507437 | 2240.107082 |
| Faecalibacterium | 0.029342637 | 0.205398459 | 51531.16746 | 66901.03939 | 68156.66663 | 49195.68509 |
| Fournierella | 0.567375938 | 0.80073217 | 83.10169694 | 211.617546 | 276.8307827 | 845.4955789 |
| Harryflintia | 0.042767426 | 0.267296412 | 82.36458675 | 114.6184086 | 217.5240073 | 344.7322513 |
| Incertae_Sedis | 0.70589107 | 0.906783602 | 22986.57514 | 20468.12022 | 20342.08675 | 18140.52516 |
| Negativibacillus | 0.819898325 | 0.927876132 | 1019.745081 | 1601.384818 | 1474.126225 | 2915.363256 |
| Paludicola | 0.573103255 | 0.802344558 | 427.2248516 | 766.8360819 | 433.8431006 | 431.1780409 |
| Phocea | 0.500784017 | 0.783818191 | 125.4017807 | 416.6153859 | 72.84733639 | 128.1251112 |
| Pygmaiobacter | 0.755977367 | 0.906783602 | 305.1247926 | 708.6976579 | 108.9497971 | 177.5646496 |
| Ruminococcus | 0.276498571 | 0.61977795 | 38921.83282 | 41471.11778 | 61945.20606 | 65469.87248 |
| Subdoligranulum | 0.158500491 | 0.504761298 | 84662.71298 | 94980.85928 | 46545.09233 | 44420.28554 |
| UBA1819 | 0.01574876 | 0.15311294 | 6607.459018 | 9353.378747 | 3370.761928 | 5439.800796 |
| Peptococcus | 0.840088878 | 0.93214418 | 147.1038176 | 444.5019402 | 657.2445312 | 2072.812043 |
| Family_XIII_AD3011_group | 0.248991299 | 0.617593186 | 2070.281133 | 1913.945462 | 1454.519356 | 1328.120674 |
| Family_XIII_UCG-001 | 0.495291616 | 0.783818191 | 206.5215952 | 208.5333556 | 308.5689503 | 402.6896064 |
| Intestinibacter | 0.326887765 | 0.640032563 | 3102.373112 | 3974.224762 | 7287.713677 | 22421.88193 |
| Paeniclostridium | 0.546217701 | 0.789984278 | 7.219424977 | 24.87763922 | 21.33129662 | 112.0529114 |
| Romboutsia | 0.374230567 | 0.667364951 | 6838.279666 | 8490.717258 | 10791.79795 | 12616.99047 |
| Terrisporobacter | 0.931551516 | 0.959496975 | 989.0093436 | 2072.979769 | 1422.054143 | 3135.570552 |
| Fenollaria | 0.811440346 | 0.927876132 | 9.404709114 | 49.66567519 | 6.631591034 | 24.06787648 |
| Acidaminococcus | 0.010974411 | 0.124415988 | 3057.179997 | 13680.45945 | 220.9479123 | 980.669349 |
| Phascolarctobacterium | 0.973756783 | 0.973756783 | 3195.702977 | 4412.342976 | 7996.361444 | 18923.16465 |
| Succiniclasticum | 0.864022265 | 0.939154636 | 149.5481878 | 661.1501742 | 125.2984904 | 747.875932 |
| Megamonas | 0.036529767 | 0.24587343 | 0 | 0 | 1043.903329 | 3935.753835 |
| Mitsuokella | 0.768221519 | 0.906783602 | 10.9682101 | 45.32002147 | 151.4410403 | 627.5790249 |
| Allisonella | 0.932794785 | 0.959496975 | 92.13412589 | 421.548197 | 46.26039404 | 207.8297957 |
| Dialister | 0.294047857 | 0.621516019 | 3601.986957 | 7545.349578 | 4006.991454 | 7072.879259 |
| Megasphaera | 0.895304231 | 0.959496975 | 982.0464885 | 3628.985411 | 732.1399247 | 3895.965361 |
| Veillonella | 0.644732998 | 0.854759657 | 97.46648527 | 223.4578207 | 235.995859 | 544.8695724 |
| Fusobacterium | 0.82037602 | 0.927876132 | 120.0071255 | 445.5673029 | 189.4174699 | 598.2170927 |
| Sneathia | 0.286868651 | 0.61977795 | 124.1089741 | 679.7728472 | 0 | 0 |
| Brevundimonas | 1.58E-07 | 2.77E-05 | 793.5791946 | 3964.718207 | 10.63230306 | 63.79381838 |
| Allorhizobium-Neorhizobium-Pararhizobium-Rhizobium | 0.286868651 | 0.61977795 | 14.74370061 | 80.75457406 | 0 | 0 |
| Mesorhizobium | 0.056289014 | 0.307830545 | 23.89751637 | 128.3045704 | 0 | 0 |
| Phyllobacterium | 0.012005457 | 0.124415988 | 128.8103589 | 698.0808112 | 0 | 0 |
| Paracoccus | 0.123710756 | 0.451070795 | 35.82170552 | 136.593043 | 0 | 0 |
| Sphingomonas | 0.025991986 | 0.189524898 | 13.82635953 | 68.75384165 | 0 | 0 |
| Succinivibrio | 0.896496229 | 0.959496975 | 36.99728416 | 202.642471 | 3.987431616 | 23.92458969 |
| Achromobacter | 0.025991986 | 0.189524898 | 39.26034004 | 201.2033446 | 0 | 0 |
| Parasutterella | 0.279852225 | 0.61977795 | 430.0922307 | 937.3007718 | 838.5939984 | 2543.920865 |
| Sutterella | 0.338795811 | 0.644448554 | 40.16856366 | 78.10523635 | 93.91463879 | 213.3180555 |
| Citrobacter | 0.086150318 | 0.396744884 | 130.8538241 | 298.2496519 | 3609.613512 | 15049.82759 |
| Enterobacter | 0.067456409 | 0.327913101 | 209.3473899 | 633.9968513 | 22.46551209 | 110.8831768 |
| Escherichia/Shigella | 0.117032189 | 0.451070795 | 4863.821202 | 8361.579102 | 15220.39345 | 29549.67182 |
| Klebsiella | 0.305837125 | 0.629664669 | 4.604214949 | 19.48688419 | 469.9655606 | 1639.488844 |
| Raoultella | 0.565040675 | 0.80073217 | 8.424772674 | 30.31894949 | 643.0903209 | 3806.977224 |
| Salmonella | 0.796239479 | 0.922794098 | 16.12190008 | 62.30195096 | 8.772090693 | 33.12590928 |
| Proteus | 0.377537887 | 0.667364951 | 0 | 0 | 1106.878561 | 6641.271365 |
| Haemophilus | 0.022629869 | 0.189524898 | 197.0844017 | 1060.842437 | 631.8641157 | 1750.792263 |
| Pseudomonas | 3.35E-06 | 0.000195305 | 2499.52389 | 13011.73732 | 225.5674551 | 1178.441687 |
| Stenotrophomonas | 0.265749125 | 0.61977795 | 145.6321462 | 506.7491175 | 2663.121217 | 14239.40241 |
| Cloacibacillus | 0.91779653 | 0.959496975 | 47.03804018 | 194.2579018 | 245.5445563 | 880.9022894 |

RRMS: relapsing-remitting multiple sclerosis

HC: healthy controls

SD: Standard Deviation

**Supplementary Table 3**: Bacteria in Yadav Dataset

| **Yadav Genera** | **p** | **q** | **MS mean** | **MS SD** | **HC mean** | **HC SD** |
| --- | --- | --- | --- | --- | --- | --- |
| Methanobrevibacter | 0.024605379 | 0.2346712 | 56.76661817 | 166.703381 | 6876.596385 | 12987.37885 |
| Methanosphaera | 0.459558525 | 0.668448763 | 0 | 0 | 23.31258513 | 133.9206057 |
| Actinomyces | 0.479019528 | 0.684313612 | 327.3361845 | 652.1401814 | 100.2112711 | 128.1358892 |
| Bifidobacterium | 0.22878371 | 0.475394721 | 21132.12003 | 33454.10865 | 29331.74187 | 38704.81524 |
| Coriobacteriaceae_UCG-002 | 0.459558525 | 0.668448763 | 0 | 0 | 19.02344147 | 109.2813513 |
| Collinsella | 0.212113962 | 0.454212059 | 16166.11499 | 15706.95239 | 10081.91704 | 13068.67347 |
| Adlercreutzia | 0.307671414 | 0.520492983 | 4577.208429 | 6373.189737 | 2200.791504 | 2401.186252 |
| DNF00809 | 0.473512081 | 0.682539937 | 237.2845815 | 449.9696334 | 94.09498011 | 143.4797982 |
| Eggerthella | 2.18E-06 | 0.000349066 | 2824.802218 | 2861.32736 | 245.893981 | 338.9851005 |
| Enterorhabdus | 0.309042709 | 0.520492983 | 116.5924425 | 521.4172543 | 110.8172096 | 299.1652722 |
| Gordonibacter | 0.045482143 | 0.293476651 | 1109.73888 | 1220.333789 | 329.6690317 | 478.9980068 |
| Senegalimassilia | 0.054630052 | 0.293476651 | 305.7249593 | 1367.243583 | 1491.223689 | 3149.141127 |
| Slackia | 0.241611436 | 0.484529449 | 1157.542766 | 3872.870588 | 845.9059241 | 1813.324047 |
| Bacteroides | 0.834382321 | 0.933574625 | 219974.2822 | 116394.4605 | 215599.536 | 116836.0581 |
| Barnesiella | 0.000181144 | 0.014491551 | 344.2301492 | 1061.295999 | 14202.026 | 20946.57848 |
| Coprobacter | 0.013930591 | 0.171453423 | 48.45819619 | 151.1890007 | 1184.585971 | 2284.113934 |
| Butyricimonas | 0.34669687 | 0.561410806 | 778.98383 | 1583.106451 | 954.8706293 | 1726.777801 |
| Odoribacter | 0.005278903 | 0.120660645 | 1284.896038 | 1861.986517 | 4440.330072 | 5755.525388 |
| CAG-873 | 0.212911903 | 0.454212059 | 959.8086314 | 4292.39469 | 0 | 0 |
| Porphyromonas | 0.156349795 | 0.454212059 | 46.87423449 | 122.7377372 | 124.4777797 | 663.500292 |
| Alloprevotella | 0.561325325 | 0.744765111 | 46.72212247 | 208.9476838 | 2299.883562 | 10897.87393 |
| Paraprevotella | 0.886783733 | 0.939674944 | 3711.385 | 11645.34291 | 4998.842895 | 13808.33088 |
| Prevotella | 0.360673621 | 0.571364152 | 6005.784799 | 21293.43912 | 37467.72577 | 70975.11066 |
| Prevotellaceae_NK3B31_group | 0.854597999 | 0.936545752 | 21.98688116 | 98.32832178 | 1405.339668 | 5751.581985 |
| Alistipes | 0.100506434 | 0.392220229 | 34244.47659 | 37498.28618 | 47355.3675 | 34140.84321 |
| Rikenellaceae_RC9_gut_group | 0.176533693 | 0.454212059 | 0 | 0 | 1010.775517 | 4900.423519 |
| Parabacteroides | 0.2551656 | 0.495097758 | 21012.85767 | 19331.05941 | 29036.56686 | 25580.20016 |
| Bilophila | 0.561519188 | 0.744765111 | 1984.377925 | 2064.566648 | 2205.795004 | 2238.727176 |
| Desulfovibrio | 0.187973762 | 0.454212059 | 6859.484922 | 24351.08102 | 2615.517303 | 5063.087399 |
| Elusimicrobium | 0.278492489 | 0.495097758 | 0 | 0 | 86.24386304 | 369.2840388 |
| Bacillus | 0.278492489 | 0.495097758 | 0 | 0 | 28.94506083 | 142.3534293 |
| Candidatus_Stoquefichus | 0.031515554 | 0.28013826 | 1707.856947 | 3607.976141 | 141.4199923 | 513.6858623 |
| Catenibacterium | 0.8283741 | 0.933379268 | 2235.902674 | 9902.132956 | 3559.774411 | 11257.41411 |
| Coprobacillus | 0.58527612 | 0.760788381 | 52.32479266 | 218.4255575 | 169.494333 | 684.205151 |
| Erysipelatoclostridium | 0.010186495 | 0.148167207 | 5306.394269 | 5501.224629 | 2714.761541 | 5843.44614 |
| Erysipelotrichaceae_UCG-003 | 0.206142685 | 0.454212059 | 16542.36684 | 20814.22545 | 21037.25855 | 20790.78273 |
| Dielma | 0.225775968 | 0.475317828 | 159.3148267 | 254.3318679 | 73.32742892 | 106.9021877 |
| Faecalicoccus | 0.212911903 | 0.454212059 | 72.29865919 | 323.3294333 | 0 | 0 |
| Faecalitalea | 0.055026872 | 0.293476651 | 4291.956971 | 7236.336835 | 3372.461508 | 8959.507974 |
| Holdemanella | 0.211355632 | 0.454212059 | 3700.735448 | 16333.86241 | 7185.412714 | 16887.63832 |
| Holdemania | 0.046446715 | 0.293476651 | 754.6583604 | 817.8320675 | 356.7552621 | 405.5521062 |
| Merdibacter | 0.096230212 | 0.392220229 | 251.9173291 | 879.3642434 | 458.3075623 | 765.382517 |
| Solobacterium | 0.04093502 | 0.293476651 | 22.57304665 | 53.33283316 | 1.610635347 | 9.252395653 |
| Turicibacter | 0.605421421 | 0.768789106 | 1032.674917 | 1400.382079 | 3019.561761 | 5954.013611 |
| Granulicatella | 0.268888799 | 0.495097758 | 100.0425125 | 251.8642654 | 15.91659461 | 34.47285061 |
| Enterococcus | 0.201078932 | 0.454212059 | 66.95574544 | 299.4351966 | 202.9317351 | 765.7376481 |
| Lactobacillus | 0.823511525 | 0.933379268 | 1245.119406 | 2711.404321 | 3578.979754 | 15562.76932 |
| Leuconostoc | 0.073305267 | 0.355419476 | 0 | 0 | 35.01308412 | 105.4307705 |
| Weissella | 0.176533693 | 0.454212059 | 0 | 0 | 56.92412486 | 186.6783044 |
| Lactococcus | 0.877519662 | 0.939674944 | 377.3076065 | 634.8276697 | 883.1835142 | 3635.497842 |
| Streptococcus | 0.113014745 | 0.420519982 | 30952.08028 | 55014.85642 | 7650.984148 | 6743.58502 |
| Gemella | 0.039234062 | 0.293476651 | 49.01613928 | 88.41327094 | 8.267209142 | 27.69611761 |
| Staphylococcus | 0.926994031 | 0.939674944 | 22.26840855 | 99.58735054 | 4.688779594 | 18.75452553 |
| Christensenella | 0.428032806 | 0.634122676 | 162.6720241 | 419.6602733 | 66.67361869 | 81.86865701 |
| Christensenellaceae_R-7_group | 0.082697839 | 0.372964316 | 9054.283469 | 13557.45546 | 15751.57503 | 17265.72734 |
| Ruminiclostridium | 0.713867494 | 0.858787963 | 48.72178613 | 103.1539781 | 164.7248091 | 415.2816829 |
| Clostridium_sensu_stricto_1 | 0.149035269 | 0.454212059 | 1939.82468 | 3486.088143 | 3554.505783 | 6077.627926 |
| Anaerofustis | 0.775189242 | 0.885930562 | 129.3668937 | 180.1423698 | 79.87463424 | 98.8218155 |
| Eubacterium | 0.68420294 | 0.839513645 | 93.41550871 | 277.5329197 | 113.7817017 | 405.407424 |
| Defluviitaleaceae_UCG-011 | 0.42463098 | 0.634122676 | 274.4911831 | 342.7249213 | 151.6718656 | 240.3474452 |
| Agathobacter | 0.506414993 | 0.716074719 | 51922.96968 | 41592.28782 | 41721.09118 | 26386.68324 |
| Anaerosporobacter | 0.278492489 | 0.495097758 | 0 | 0 | 39.30970529 | 199.1345714 |
| Anaerostipes | 0.097049 | 0.392220229 | 32315.30116 | 45248.49513 | 18611.48444 | 14062.05604 |
| Blautia | 0.005030254 | 0.120660645 | 115398.1286 | 66531.47249 | 71849.42331 | 42941.16054 |
| CAG-56 | 0.147710708 | 0.454212059 | 1166.599249 | 3469.950586 | 1231.618917 | 1971.471147 |
| Coprococcus | 0.626540101 | 0.789341859 | 13110.17285 | 17336.60737 | 14796.89713 | 16495.54275 |
| Dorea | 0.920355022 | 0.939674944 | 18318.51716 | 22350.56126 | 14331.89844 | 11550.86301 |
| Eisenbergiella | 0.550620564 | 0.744765111 | 553.794618 | 1010.901439 | 374.0015234 | 716.6181706 |
| FD2005 | 0.212911903 | 0.454212059 | 267.7428624 | 1197.382481 | 0 | 0 |
| Frisingicoccus | 0.425048628 | 0.634122676 | 488.2591099 | 843.7003724 | 896.8366663 | 2094.159714 |
| Fusicatenibacter | 0.693179574 | 0.840217666 | 10578.62138 | 8946.043083 | 10695.28772 | 7470.986398 |
| GCA-900066575 | 0.598306257 | 0.765832009 | 384.4370322 | 530.185119 | 426.0071722 | 649.9002651 |
| GCA-900066755 | 0.988248341 | 0.988248341 | 16.45747829 | 42.76543596 | 33.75817655 | 129.5814574 |
| Howardella | 0.925050003 | 0.939674944 | 333.9463631 | 840.9631157 | 380.006275 | 1132.020356 |
| Hungatella | 0.003700729 | 0.118423342 | 298.1135882 | 733.0329241 | 45.72777638 | 115.1763458 |
| Lachnoclostridium | 0.036461349 | 0.293476651 | 17712.07384 | 13301.43215 | 10066.58053 | 7912.680085 |
| Lachnospira | 0.023430992 | 0.2346712 | 4865.241845 | 7652.031545 | 8951.983304 | 9939.40637 |
| Lachnospiraceae_AC2044_group | 0.170053701 | 0.454212059 | 30.82941764 | 137.8733471 | 617.9911813 | 1804.265713 |
| Lachnospiraceae_FCS020_group | 0.008171652 | 0.132833323 | 818.8048626 | 1894.349281 | 1387.6011 | 1645.04893 |
| Lachnospiraceae_ND3007_group | 0.38227492 | 0.588115262 | 3431.394189 | 6561.139049 | 3682.623751 | 4737.597847 |
| Lachnospiraceae_NK4A136_group | 0.079589265 | 0.372964316 | 4414.800416 | 5394.854362 | 7800.842859 | 7772.909268 |
| Lachnospiraceae_NK4B4_group | 0.773412376 | 0.885930562 | 189.0988452 | 409.6656889 | 316.6779086 | 995.8435536 |
| Lachnospiraceae_UCG-001 | 0.184095691 | 0.454212059 | 1439.915616 | 4442.538693 | 1198.513364 | 2413.110192 |
| Lachnospiraceae_UCG-002 | 0.302630415 | 0.520492983 | 646.9207834 | 2804.439967 | 16.28403072 | 93.54463459 |
| Lachnospiraceae_UCG-003 | 0.561325325 | 0.744765111 | 13.1114473 | 58.6361749 | 485.3070867 | 1854.62604 |
| Lachnospiraceae_UCG-004 | 0.042070137 | 0.293476651 | 149.5162868 | 318.9504301 | 494.3255488 | 768.4512548 |
| Lachnospiraceae_UCG-010 | 0.148760177 | 0.454212059 | 640.1651742 | 1075.34775 | 564.3470866 | 421.1722439 |
| Lactonifactor | 0.242264725 | 0.484529449 | 62.12830314 | 145.9466568 | 18.78344901 | 69.4927275 |
| Marvinbryantia | 0.024933815 | 0.2346712 | 2481.476946 | 5189.256498 | 2258.53814 | 2153.116226 |
| Roseburia | 0.848604794 | 0.936391497 | 11427.66837 | 10150.61394 | 11588.84723 | 13983.71531 |
| Sellimonas | 0.133029835 | 0.454212059 | 35.26559205 | 107.9756435 | 18.69362287 | 107.3866877 |
| Shuttleworthia | 0.372181078 | 0.578145364 | 253.9838365 | 557.954141 | 223.2044712 | 366.6388503 |
| Tuzzerella | 0.132148854 | 0.454212059 | 1385.650322 | 2836.165748 | 707.3019214 | 1812.815675 |
| Tyzzerella | 0.347372936 | 0.561410806 | 276.870457 | 1132.410368 | 443.4009229 | 1989.451641 |
| UC5-1-2E3 | 0.747011374 | 0.878836911 | 33.80376281 | 92.57575001 | 55.56451575 | 172.2951841 |
| Monoglobus | 0.185120061 | 0.454212059 | 9228.949345 | 8144.935877 | 5409.956766 | 3661.309619 |
| Butyricicoccus | 0.354072331 | 0.56651573 | 7902.167269 | 8975.95874 | 5026.228987 | 5140.779419 |
| UCG-008 | 0.927929007 | 0.939674944 | 147.0718593 | 552.403328 | 109.1828173 | 394.8584388 |
| UCG-009 | 0.022946094 | 0.2346712 | 20.02480903 | 62.41980536 | 112.5211612 | 193.6310919 |
| Acetanaerobacterium | 0.007656467 | 0.132833323 | 54.7260218 | 66.45457821 | 15.85083613 | 41.73694223 |
| Colidextribacter | 0.2399184 | 0.484529449 | 1716.219728 | 1875.422696 | 1795.798799 | 1054.510252 |
| Flavonifractor | 0.332572305 | 0.548572873 | 1037.540574 | 1278.748159 | 677.1840464 | 1214.989502 |
| Intestinimonas | 0.589610996 | 0.760788381 | 1355.797817 | 2752.995366 | 638.4694604 | 1295.183262 |
| NK4A214_group | 0.083916971 | 0.372964316 | 3626.403028 | 6581.527535 | 5034.28497 | 6690.590383 |
| Oscillibacter | 0.255251834 | 0.495097758 | 2126.722 | 3619.566152 | 3202.319639 | 5097.546738 |
| Oscillospira | 0.136366427 | 0.454212059 | 80.05331212 | 222.558246 | 118.4385985 | 190.3797682 |
| Pseudoflavonifractor | 0.645018711 | 0.806273388 | 13.08043325 | 49.97700677 | 15.82581876 | 69.08323445 |
| UCG-002 | 0.008302083 | 0.132833323 | 6497.503884 | 10395.01457 | 20061.37813 | 21609.94538 |
| UCG-003 | 0.000730455 | 0.0389576 | 171.8612555 | 423.3034892 | 1267.328868 | 1792.446713 |
| UCG-005 | 0.100465286 | 0.392220229 | 4088.820227 | 8691.84905 | 4089.141327 | 5358.705596 |
| Hydrogenoanaerobacterium | 0.054630052 | 0.293476651 | 16.98369565 | 75.95339597 | 54.49244026 | 119.5211516 |
| Anaerofilum | 0.557737809 | 0.744765111 | 73.60724913 | 98.67128404 | 91.85291431 | 120.4758897 |
| Anaerotruncus | 0.580803514 | 0.760788381 | 149.643812 | 229.8940614 | 264.9996598 | 596.9431027 |
| Angelakisella | 0.927929007 | 0.939674944 | 11.28736722 | 41.56869934 | 11.57662712 | 39.14659319 |
| CAG-352 | 0.365726212 | 0.573688175 | 143.152537 | 640.1976077 | 1089.458209 | 3329.907063 |
| Candidatus_Soleaferrea | 0.070497847 | 0.352489237 | 262.9506547 | 255.1505093 | 147.5750471 | 162.8698512 |
| Caproiciproducens | 0.161264907 | 0.454212059 | 154.6585322 | 531.7851864 | 99.31558663 | 236.0042454 |
| DTU089 | 0.146700635 | 0.454212059 | 543.2601998 | 720.7333339 | 346.7660614 | 667.0011016 |
| Faecalibacterium | 0.183373838 | 0.454212059 | 68580.44173 | 69931.5267 | 80294.92605 | 39057.61033 |
| Fournierella | 0.150435355 | 0.454212059 | 8.759773158 | 28.35774224 | 169.3976219 | 481.2619102 |
| Incertae_Sedis | 0.277661669 | 0.495097758 | 16081.48244 | 21528.07494 | 8642.144344 | 8530.124812 |
| Negativibacillus | 0.92543218 | 0.939674944 | 1686.78859 | 2178.788627 | 1873.139836 | 3012.999665 |
| Paludicola | 0.842306946 | 0.935896607 | 265.8352743 | 481.4348755 | 429.0409485 | 998.5811271 |
| Phocea | 0.736093556 | 0.876717913 | 57.83689252 | 97.25208811 | 40.67903003 | 69.21247317 |
| Pygmaiobacter | 0.510203237 | 0.716074719 | 46.82240404 | 87.97621348 | 46.95443302 | 125.9247217 |
| Ruminococcus | 0.195612135 | 0.454212059 | 30386.66592 | 37178.15621 | 35327.3181 | 25149.31952 |
| Subdoligranulum | 0.532624126 | 0.741042263 | 21894.74318 | 21621.44249 | 22899.37767 | 17783.54751 |
| UBA1819 | 0.048527569 | 0.293476651 | 1522.885344 | 1867.829497 | 622.4099851 | 694.2763026 |
| Peptococcus | 0.770597487 | 0.885930562 | 1144.60534 | 3473.619043 | 70.68259787 | 235.4872358 |
| Family_XIII_AD3011_group | 0.563228615 | 0.744765111 | 3292.453448 | 4109.842015 | 1879.948983 | 1552.232331 |
| Family_XIII_UCG-001 | 0.061666029 | 0.318276278 | 378.082836 | 456.4035844 | 629.7985165 | 573.446149 |
| Intestinibacter | 0.687351797 | 0.839513645 | 1500.182063 | 2586.456607 | 1201.830391 | 2142.356615 |
| Romboutsia | 0.876046859 | 0.939674944 | 7731.670071 | 12454.22358 | 5933.01226 | 6491.313462 |
| Terrisporobacter | 0.298018565 | 0.518293157 | 148.2503779 | 361.1326981 | 289.5894755 | 516.8718136 |
| Anaerococcus | 0.674023128 | 0.835997678 | 6.726601085 | 21.49270724 | 14.23859265 | 62.53114095 |
| Acidaminococcus | 0.988248341 | 0.988248341 | 1418.623451 | 6195.918072 | 537.5249588 | 1727.03422 |
| Phascolarctobacterium | 0.3272879 | 0.545479834 | 11402.1826 | 13313.43915 | 13400.42229 | 12812.60362 |
| Succiniclasticum | 0.176533693 | 0.454212059 | 0 | 0 | 1924.109683 | 7704.818479 |
| Megamonas | 0.176533693 | 0.454212059 | 0 | 0 | 3053.2131 | 14891.50015 |
| Mitsuokella | 0.278492489 | 0.495097758 | 0 | 0 | 87.32876403 | 373.0899433 |
| Allisonella | 0.104708874 | 0.398890948 | 226.8578767 | 694.9749251 | 9.953997219 | 57.18136061 |
| Dialister | 0.863148025 | 0.939480844 | 6024.239001 | 11935.42056 | 6024.666243 | 9503.015442 |
| Megasphaera | 0.129137671 | 0.454212059 | 5860.321076 | 20863.18485 | 1010.858577 | 5507.78354 |
| Veillonella | 0.92062957 | 0.939674944 | 292.2099861 | 779.8949487 | 225.8372512 | 549.7632236 |
| Fusobacterium | 0.048629364 | 0.293476651 | 1206.314753 | 5244.062166 | 23.75275212 | 136.4491726 |
| TM7x | 0.002496031 | 0.099841255 | 70.01476989 | 170.4519596 | 5.974821571 | 26.6660304 |
| Succinivibrio | 0.176533693 | 0.454212059 | 0 | 0 | 286.248533 | 1037.582259 |
| Ralstonia | 0.201343999 | 0.454212059 | 165.6858168 | 220.9570917 | 220.2308252 | 634.9574073 |
| Comamonas | 0.278492489 | 0.495097758 | 0 | 0 | 31.86962398 | 134.1712293 |
| Oxalobacter | 0.086692029 | 0.374884451 | 85.03567664 | 338.0079576 | 112.4721092 | 249.1945978 |
| Parasutterella | 0.39839849 | 0.607083413 | 2751.554172 | 5396.437311 | 4048.643635 | 6962.018772 |
| Sutterella | 0.053917605 | 0.293476651 | 1063.108542 | 2647.400619 | 3799.061694 | 8296.206025 |
| Citrobacter | 0.278492489 | 0.495097758 | 0 | 0 | 30.94494213 | 147.7189041 |
| Enterobacter | 0.012054867 | 0.160731557 | 0 | 0 | 1106.180837 | 5184.289604 |
| Escherichia/Shigella | 0.293002171 | 0.515168652 | 6457.819666 | 16624.06628 | 8824.328033 | 42390.11869 |
| Klebsiella | 0.176533693 | 0.454212059 | 0 | 0 | 603.6865446 | 3411.355952 |
| Haemophilus | 0.912126417 | 0.939674944 | 489.0707477 | 1156.464499 | 462.4585795 | 894.2120167 |
| Pseudomonas | 0.759861724 | 0.885930562 | 8.073072842 | 36.10387932 | 69.69191241 | 400.3495568 |
| Cloacibacillus | 0.176533693 | 0.454212059 | 0 | 0 | 27.04737619 | 90.63922892 |
| Akkermansia | 0.739730739 | 0.876717913 | 46878.00745 | 65230.53107 | 26520.42643 | 40971.31417 |

RRMS: relapsing-remitting multiple sclerosis

HC: healthy controls

SD: Standard Deviation

**Supplementary Table 4**: Significant Pathways in Chen Dataset

| **Chen Pathways** | **p** | **q** | **RRMS mean** | **RRMS**  **SD** | **HC**  **mean** | **HC**  **SD** |
| --- | --- | --- | --- | --- | --- | --- |
| 3-HYDROXYPHENYLACETATE-DEGRADATION-PWY | 0.076439054 | 0.192014903 | 18.21781099 | 23.66209456 | 89.78755157 | 213.8361569 |
| AEROBACTINSYN-PWY | 0.085940345 | 0.205994414 | 2.719609308 | 6.423659556 | 34.69965673 | 148.4162589 |
| ALL-CHORISMATE-PWY | 0.041195598 | 0.146316011 | 97.51540513 | 115.8990517 | 283.7561328 | 483.5426347 |
| ARG+POLYAMINE-SYN | 0.002971773 | 0.036403356 | 720.3516895 | 490.6548872 | 1164.347683 | 690.3022332 |
| ASPASN-PWY | 0.112142808 | 0.241183848 | 6582.932017 | 608.7012167 | 6777.745786 | 644.6167641 |
| BIOTIN-BIOSYNTHESIS-PWY | 0.106380729 | 0.23523626 | 2211.664286 | 1405.772276 | 2731.436388 | 1310.1212 |
| BRANCHED-CHAIN-AA-SYN-PWY | 0.098174548 | 0.221775597 | 8951.512732 | 690.8528885 | 8540.851382 | 992.2075526 |
| CALVIN-PWY | 0.109232157 | 0.238186787 | 9125.953405 | 606.9244728 | 8889.900049 | 1011.890071 |
| COA-PWY | 0.095552754 | 0.220614447 | 7021.081233 | 751.4743479 | 7357.969324 | 843.0560173 |
| COLANSYN-PWY | 0.008826654 | 0.065989745 | 3632.599386 | 690.7164069 | 4051.205762 | 987.6617927 |
| DENOVOPURINE2-PWY | 0.044712978 | 0.146316011 | 4653.881488 | 1260.218228 | 5188.568278 | 1360.812347 |
| ECASYN-PWY | 0.027150428 | 0.120073723 | 30.02058321 | 42.7668895 | 136.2458369 | 294.9103436 |
| ENTBACSYN-PWY | 0.052560774 | 0.155698896 | 68.21501415 | 91.07321586 | 229.5760645 | 418.8900641 |
| FASYN-ELONG-PWY | 0.041984619 | 0.146316011 | 4482.458709 | 1799.298197 | 5443.81837 | 2142.324418 |
| FUC-RHAMCAT-PWY | 3.49E-05 | 0.002189593 | 2065.863702 | 477.6323656 | 1543.575669 | 461.7998328 |
| FUCCAT-PWY | 1.82E-05 | 0.001428107 | 2270.830486 | 958.3860502 | 1377.469492 | 662.1804771 |
| GALACT-GLUCUROCAT-PWY | 0.007836337 | 0.061515247 | 2727.620567 | 508.4415324 | 2341.575807 | 765.1427412 |
| GALACTUROCAT-PWY | 0.062477185 | 0.169119277 | 3791.442727 | 743.1951475 | 3355.206248 | 790.8068615 |
| GLCMANNANAUT-PWY | 0.003106425 | 0.036403356 | 3750.10279 | 837.779543 | 3122.481766 | 839.5603042 |
| GLUCOSE1PMETAB-PWY | 0.079877422 | 0.195556913 | 74.56602154 | 169.8293571 | 204.2246467 | 392.6103663 |
| GLUCUROCAT-PWY | 0.028373494 | 0.120395637 | 2699.101749 | 519.7840566 | 2387.17021 | 857.1546772 |
| GLYCOCAT-PWY | 0.030339696 | 0.125350851 | 10216.17999 | 1103.843136 | 9621.92866 | 1021.088294 |
| GLYCOL-GLYOXDEG-PWY | 0.079877422 | 0.195556913 | 35.02399535 | 41.11350092 | 106.6384307 | 193.8465632 |
| GLYCOLYSIS-E-D | 0.043330922 | 0.146316011 | 2622.374199 | 971.4521271 | 2138.990213 | 897.0672148 |
| GLYOXYLATE-BYPASS | 0.113197196 | 0.241795371 | 74.29264524 | 210.4580606 | 194.1490695 | 388.3234181 |
| HEME-BIOSYNTHESIS-II | 0.058876472 | 0.162168528 | 154.0172574 | 234.383186 | 235.6244778 | 281.1530799 |
| HEXITOLDEGSUPER-PWY | 0.080963053 | 0.195556913 | 1682.691921 | 584.7257604 | 1440.264059 | 603.6315548 |
| ILEUSYN-PWY | 0.049080629 | 0.151091348 | 9541.623199 | 683.1438361 | 9054.930618 | 1032.067198 |
| KDO-NAGLIPASYN-PWY | 0.0078086 | 0.061515247 | 102.3068069 | 129.4670855 | 345.1478307 | 542.6163226 |
| LACTOSECAT-PWY | 0.015046953 | 0.087495243 | 303.3520207 | 149.6409431 | 229.8750561 | 128.9607334 |
| LEU-DEG2-PWY | 0.005271727 | 0.051647505 | 68.63779745 | 221.4023178 | 43.8914366 | 167.7647159 |
| LPSSYN-PWY | 0.02072728 | 0.102595389 | 40.09370467 | 63.27815441 | 140.3752286 | 246.7260432 |
| METH-ACETATE-PWY | 9.33E-05 | 0.003256701 | 3240.890834 | 1273.503418 | 2088.251752 | 1132.77716 |
| NAGLIPASYN-PWY | 0.033504566 | 0.126752214 | 1973.710758 | 1095.306317 | 2609.717575 | 1363.311216 |
| P105-PWY | 0.11764932 | 0.244061951 | 266.9169056 | 543.7094894 | 626.8932289 | 1011.938385 |
| P161-PWY | 0.078714654 | 0.195556913 | 6142.10045 | 1396.960626 | 5553.44691 | 1512.443451 |
| P163-PWY | 0.115113398 | 0.242587967 | 264.6342551 | 203.2904085 | 213.559612 | 218.9822799 |
| P164-PWY | 0.011576246 | 0.078661341 | 2831.378864 | 1413.227402 | 2164.814997 | 897.6434532 |
| P221-PWY | 0.005427922 | 0.051647505 | 845.62529 | 527.9842588 | 532.1456087 | 382.9724193 |
| P281-PWY | 0.067550109 | 0.178241463 | 10.04316844 | 40.83299113 | 12.19017882 | 58.21726454 |
| P341-PWY | 0.023925512 | 0.108878415 | 98.66722252 | 88.28271749 | 65.13715182 | 82.55130499 |
| P381-PWY | 1.71E-05 | 0.001428107 | 21.96475074 | 85.05060734 | 0.348622688 | 2.091736127 |
| P42-PWY | 0.090475149 | 0.213602984 | 5428.19757 | 2063.620352 | 6227.72726 | 2268.093481 |
| P441-PWY | 0.068212936 | 0.178490517 | 3298.94474 | 974.5065433 | 2875.122647 | 901.3640182 |
| P461-PWY | 0.020087483 | 0.102595389 | 2069.862047 | 1144.175708 | 1499.956324 | 945.2220244 |
| P562-PWY | 0.001886793 | 0.028212046 | 796.2800612 | 544.4725939 | 454.8372141 | 323.4135721 |
| PANTO-PWY | 0.010317509 | 0.074823161 | 4240.287658 | 887.1561371 | 4782.20064 | 772.9313329 |
| PANTOSYN-PWY | 0.006402359 | 0.057438302 | 4860.308446 | 869.5559561 | 5419.591465 | 735.0306542 |
| POLYAMINSYN3-PWY | 0.095552754 | 0.220614447 | 310.2315872 | 240.161115 | 385.2114574 | 223.7593489 |
| POLYAMSYN-PWY | 0.001639822 | 0.025745203 | 402.2133195 | 305.0060843 | 718.6686541 | 504.471659 |
| POLYISOPRENSYN-PWY | 0.066255406 | 0.176306758 | 3894.798543 | 1248.683834 | 4448.476512 | 1400.125912 |
| PPGPPMET-PWY | 0.01269858 | 0.081374575 | 83.58451245 | 227.8985824 | 234.6307108 | 448.6905462 |
| PROTOCATECHUATE-ORTHO-CLEAVAGE-PWY | 0.003130225 | 0.036403356 | 32.68797078 | 166.6521788 | 4.951821596 | 16.00583134 |
| PRPP-PWY | 0.031365288 | 0.126752214 | 1400.215806 | 916.7587804 | 1866.195407 | 940.1656 |
| PWY-1269 | 0.018048854 | 0.09771276 | 1983.152922 | 1035.630588 | 2688.923237 | 1321.620751 |
| PWY-1861 | 0.008484957 | 0.064982354 | 3068.41626 | 1377.605512 | 2212.81526 | 1305.891387 |
| PWY-2941 | 0.112142808 | 0.241183848 | 1051.183632 | 730.4789666 | 1382.598979 | 878.664103 |
| PWY-4984 | 0.018707608 | 0.099562522 | 853.9919792 | 535.0737892 | 1368.464437 | 876.7131264 |
| PWY-5022 | 0.003701482 | 0.040457342 | 179.5870341 | 138.1292055 | 293.6894787 | 192.8029277 |
| PWY-5101 | 0.055447366 | 0.161208081 | 10333.66857 | 774.3929531 | 9766.291911 | 1138.215031 |
| PWY-5104 | 0.033504566 | 0.126752214 | 9934.886327 | 732.0808488 | 9386.105859 | 1231.203219 |
| PWY-5177 | 0.115113398 | 0.242587967 | 112.0726679 | 103.5931793 | 188.7763305 | 196.9040917 |
| PWY-5180 | 0.020911162 | 0.102595389 | 47.78624702 | 62.73135901 | 26.95294286 | 42.04620708 |
| PWY-5181 | 0.005789787 | 0.053470383 | 18.44300927 | 94.70229996 | 2.515305349 | 6.92890064 |
| PWY-5182 | 0.020911162 | 0.102595389 | 47.78624702 | 62.73135901 | 26.95294286 | 42.04620708 |
| PWY-5188 | 0.018048854 | 0.09771276 | 3153.974238 | 787.3172104 | 2696.403515 | 718.3549701 |
| PWY-5189 | 0.002842386 | 0.036403356 | 2983.057804 | 842.341251 | 2420.62027 | 694.5587669 |
| PWY-5304 | 0.000472647 | 0.010209445 | 1766.091411 | 725.9293374 | 1183.882421 | 737.8356903 |
| PWY-5384 | 4.78E-05 | 0.002258045 | 5481.238369 | 1622.255941 | 3893.424762 | 1254.506159 |
| PWY-5415 | 0.010499948 | 0.074823161 | 18.16945129 | 40.37008178 | 6.78888821 | 12.10376484 |
| PWY-5505 | 0.046131481 | 0.146316011 | 6563.488326 | 1411.531309 | 5552.649663 | 1930.566024 |
| PWY-5507 | 0.007243548 | 0.061515247 | 37.05815458 | 74.12577598 | 50.67491825 | 188.2623183 |
| PWY-5659 | 0.106380729 | 0.23523626 | 5534.43831 | 1084.82882 | 5880.306646 | 930.1018773 |
| PWY-5705 | 0.044712978 | 0.146316011 | 301.7763454 | 181.9651139 | 221.876006 | 143.8394184 |
| PWY-5747 | 0.035238539 | 0.130175308 | 31.19659391 | 96.27891844 | 71.02750585 | 136.8480352 |
| PWY-5855 | 0.058876472 | 0.162168528 | 89.35906063 | 206.906134 | 203.7749623 | 348.2356294 |
| PWY-5856 | 0.058876472 | 0.162168528 | 89.35906063 | 206.906134 | 203.7749623 | 348.2356294 |
| PWY-5857 | 0.058876472 | 0.162168528 | 89.35906063 | 206.906134 | 203.7749623 | 348.2356294 |
| PWY-5910 | 0.046131481 | 0.146316011 | 61.30240812 | 74.29215314 | 147.8224967 | 234.0513533 |
| PWY-5913 | 0.034619519 | 0.12941106 | 1740.156743 | 1068.142341 | 2232.934894 | 1040.556504 |
| PWY-5918 | 0.062477185 | 0.169119277 | 325.3272144 | 411.1424337 | 446.2477528 | 424.3665986 |
| PWY-5941 | 1.26E-08 | 3.94E-06 | 20.05482047 | 100.2769912 | 0 | 0 |
| PWY-5971 | 0.050612686 | 0.152811379 | 1543.850757 | 1056.503116 | 2149.478221 | 1291.199937 |
| PWY-5973 | 0.058876472 | 0.162168528 | 8872.719892 | 895.0704165 | 9384.596366 | 909.7325508 |
| PWY-6071 | 0.108860697 | 0.238186787 | 8.298076309 | 20.83562722 | 50.65423901 | 204.6417076 |
| PWY-6122 | 0.098174548 | 0.221775597 | 8952.400127 | 662.0035424 | 8607.878536 | 885.5088861 |
| PWY-6125 | 0.043330922 | 0.146316011 | 3679.137787 | 1219.725872 | 4276.107557 | 1411.18858 |
| PWY-621 | 0.00206963 | 0.029539266 | 8594.337608 | 1722.323462 | 7079.279556 | 1902.343838 |
| PWY-6277 | 0.098174548 | 0.221775597 | 8952.400127 | 662.0035424 | 8607.878536 | 885.5088861 |
| PWY-6353 | 0.000111451 | 0.003499568 | 3636.90394 | 775.4882893 | 2834.671753 | 749.5311951 |
| PWY-6467 | 0.033504566 | 0.126752214 | 1759.284931 | 982.7776338 | 2324.819011 | 1202.670291 |
| PWY-6519 | 0.118144639 | 0.244061951 | 2212.75252 | 1551.578715 | 2780.853763 | 1455.389125 |
| PWY-6545 | 0.032420035 | 0.126752214 | 2266.624166 | 623.8060697 | 2627.562422 | 792.661115 |
| PWY-6608 | 0.000382392 | 0.009236244 | 3861.916936 | 1111.385269 | 2966.168067 | 680.9321738 |
| PWY-6628 | 0.003106425 | 0.036403356 | 2041.054179 | 1043.690734 | 3005.27783 | 1347.717567 |
| PWY-6629 | 0.017462574 | 0.09771276 | 117.0780481 | 189.1448921 | 381.9972583 | 621.1329157 |
| PWY-6630 | 0.003865351 | 0.040457342 | 2039.5076 | 1045.055514 | 2988.050128 | 1337.547016 |
| PWY-6700 | 0.050612686 | 0.152811379 | 3901.653189 | 1086.537139 | 4389.245807 | 1137.121704 |
| PWY-6703 | 0.01450158 | 0.085915024 | 2840.001585 | 736.5779576 | 3403.046899 | 1015.969397 |
| PWY-6708 | 0.058876472 | 0.162168528 | 89.35906063 | 206.906134 | 203.7749623 | 348.2356294 |
| PWY-6892 | 0.052184015 | 0.155698896 | 5401.485948 | 1052.588855 | 4994.815942 | 908.6224188 |
| PWY-6969 | 0.029342637 | 0.12284784 | 4216.784628 | 1489.96508 | 4890.317456 | 1452.704207 |
| PWY-7003 | 0.001173392 | 0.019391846 | 1226.315058 | 762.7739688 | 725.5554713 | 540.8256787 |
| PWY-7013 | 0.07436816 | 0.188319373 | 310.5178528 | 372.9501884 | 220.4113322 | 371.767818 |
| PWY-7111 | 0.000524757 | 0.010209445 | 11981.27869 | 1673.311946 | 10387.60097 | 1722.558863 |
| PWY-7184 | 0.032420035 | 0.126752214 | 2567.705144 | 777.5368907 | 3030.351684 | 1019.903655 |
| PWY-7187 | 0.046131481 | 0.146316011 | 3606.645041 | 596.4160819 | 3900.357585 | 818.5867153 |
| PWY-7196 | 0.033504566 | 0.126752214 | 3679.042597 | 1364.610667 | 4337.837526 | 1535.877257 |
| PWY-7197 | 0.019386979 | 0.101458525 | 2372.079244 | 946.4236553 | 2872.880636 | 1061.893131 |
| PWY-7198 | 5.75E-05 | 0.002258045 | 1379.399945 | 346.431088 | 994.7363764 | 442.3773141 |
| PWY-7200 | 0.021553978 | 0.104122294 | 2839.666765 | 810.2769089 | 3266.246785 | 904.2412864 |
| PWY-7210 | 5.08E-05 | 0.002258045 | 1615.520721 | 335.7770244 | 1205.066313 | 466.1191389 |
| PWY-7220 | 0.000552741 | 0.010209445 | 10869.48322 | 1458.022641 | 9661.515852 | 1319.583008 |
| PWY-7222 | 0.000552741 | 0.010209445 | 10869.48322 | 1458.022641 | 9661.515852 | 1319.583008 |
| PWY-7228 | 0.038153382 | 0.137703011 | 3325.943008 | 1164.741225 | 3932.363614 | 1377.819537 |
| PWY-7234 | 0.013462145 | 0.08454227 | 506.9059765 | 558.3554087 | 827.3357221 | 724.8144796 |
| PWY-7237 | 0.000964604 | 0.016826977 | 4182.359703 | 2958.024584 | 2370.356631 | 1281.9752 |
| PWY-7242 | 0.025627522 | 0.114957744 | 5163.019302 | 866.231212 | 4714.912212 | 894.001426 |
| PWY-7315 | 0.000125276 | 0.003576072 | 2401.462849 | 1041.29646 | 1500.17568 | 812.6367116 |
| PWY-7323 | 0.044712978 | 0.146316011 | 3639.312202 | 1020.724182 | 4131.365175 | 1321.273545 |
| PWY-7328 | 0.106380729 | 0.23523626 | 717.67908 | 491.5945629 | 988.5261245 | 648.4254225 |
| PWY-7376 | 1.71E-05 | 0.001428107 | 22.41281784 | 100.890883 | 0.207693964 | 1.246163782 |
| PWY-7431 | 0.005191284 | 0.051647505 | 38.12616654 | 157.388753 | 5.578642583 | 15.38901825 |
| PWY-7446 | 0.016279619 | 0.092941827 | 10.46997909 | 17.21060881 | 41.30359455 | 78.55162881 |
| PWY-7456 | 0.012024664 | 0.078661341 | 1847.621226 | 878.2466872 | 2422.606706 | 905.0026951 |
| PWY-7663 | 0.046131481 | 0.146316011 | 9389.615592 | 854.0427631 | 9869.353932 | 749.2684908 |
| PWY-841 | 0.038153382 | 0.137703011 | 4510.628844 | 1245.146191 | 5069.840463 | 1363.739049 |
| PWY-922 | 0.04758713 | 0.149423589 | 43.17870995 | 52.5968367 | 105.7675356 | 170.3570746 |
| PWY0-1061 | 0.013973461 | 0.085915024 | 3583.911449 | 1105.303654 | 2858.165212 | 1014.982677 |
| PWY0-1241 | 0.066255406 | 0.176306758 | 324.1629507 | 202.0700079 | 447.9628525 | 290.9569475 |
| PWY0-1296 | 0.003865351 | 0.040457342 | 6507.021876 | 1294.35031 | 5498.520597 | 1244.929417 |
| PWY0-1297 | 0.01450158 | 0.085915024 | 5772.423627 | 1152.143351 | 5025.153019 | 1170.768575 |
| PWY0-1298 | 0.007836337 | 0.061515247 | 5801.558751 | 1100.167048 | 4999.806173 | 1107.347 |
| PWY0-1338 | 0.028058624 | 0.120395637 | 23.74242016 | 30.96526361 | 100.2338212 | 214.0168326 |
| PWY0-1479 | 0.022321038 | 0.106194029 | 146.4513741 | 147.5475096 | 315.5997649 | 392.1181446 |
| PWY0-162 | 0.023925512 | 0.108878415 | 4233.057949 | 1313.31126 | 4854.444909 | 1432.278248 |
| PWY0-166 | 0.028373494 | 0.120395637 | 3463.61904 | 799.8677935 | 3849.729849 | 885.3855448 |
| PWY0-42 | 0.023024682 | 0.107906718 | 21.32638405 | 46.5386073 | 69.81888343 | 133.0550122 |
| PWY0-781 | 0.088017854 | 0.209375805 | 2265.035396 | 550.6995553 | 2591.103772 | 743.6110197 |
| PWY0-845 | 0.080963053 | 0.195556913 | 1562.037699 | 859.1367943 | 1963.535217 | 1080.048017 |
| PYRIDOXSYN-PWY | 0.095552754 | 0.220614447 | 1215.093794 | 743.9121647 | 1566.157564 | 964.8434139 |
| REDCITCYC | 0.012024664 | 0.078661341 | 1279.572735 | 1213.029334 | 836.4975519 | 818.4194576 |
| RIBOSYN2-PWY | 0.070217074 | 0.182216209 | 4926.225967 | 772.6740173 | 5270.978493 | 757.4398242 |
| RUMP-PWY | 0.010723064 | 0.074823161 | 1626.693673 | 576.561823 | 1268.550518 | 688.5510042 |
| SALVADEHYPOX-PWY | 0.000343476 | 0.008987614 | 3545.860668 | 1258.391151 | 2479.695033 | 930.2953487 |
| SO4ASSIM-PWY | 0.07436816 | 0.188319373 | 1256.175308 | 548.6622479 | 1523.198734 | 611.0468937 |
| SULFATE-CYS-PWY | 0.07436816 | 0.188319373 | 2283.844587 | 854.7788583 | 2675.3764 | 910.847373 |
| TCA | 0.046131481 | 0.146316011 | 2865.818097 | 1323.937645 | 3455.36963 | 1208.66564 |
| THISYN-PWY | 0.118144639 | 0.244061951 | 3579.546708 | 1111.955905 | 4000.17637 | 1178.384891 |
| TYRFUMCAT-PWY | 0.007725081 | 0.061515247 | 48.28022076 | 187.9881684 | 25.18697978 | 122.00505 |
| UBISYN-PWY | 0.055447366 | 0.161208081 | 87.21772318 | 202.487806 | 198.1901446 | 336.8681978 |
| VALSYN-PWY | 0.049080629 | 0.151091348 | 9541.623199 | 683.1438361 | 9054.930618 | 1032.067198 |

RRMS: relapsing-remitting multiple sclerosis

HC: healthy controls

SD: Standard Deviation

**Supplementary Table 5**: Significant Pathways in Yadav Dataset

| **Yadav Pathways** | **p** | **q** | **RRMS mean** | **RRMS**  **SD** | **HC**  **mean** | **HC**  **SD** |
| --- | --- | --- | --- | --- | --- | --- |
| biotin biosynthesis I | 0.215426045 | 0.045809932 | 2223.018926 | 726.8241009 | 2712.881157 | 1005.425058 |
| Calvin-Benson-Bassham cycle | 0.121557301 | 0.006397753 | 8900.46916 | 339.1519459 | 8569.490434 | 480.4598937 |
| colanic acid building blocks biosynthesis | 0.211247264 | 0.041857043 | 3404.568466 | 458.2780597 | 3689.245316 | 596.2297982 |
| fatty acid elongation -- saturated | 0.211247264 | 0.039989303 | 4307.681672 | 1196.49234 | 5000.343883 | 1059.263552 |
| GLCMANNANAUT-PWY | 0.029910124 | 0.000185202 | 3819.216136 | 665.2518131 | 3127.190955 | 579.1698487 |
| GLYCOCAT-PWY | 0.180625849 | 0.020321024 | 10099.67867 | 767.5572657 | 9637.614928 | 662.7869464 |
| L-fucose degradation I | 0.111825499 | 0.00346209 | 2085.083315 | 716.7707646 | 1557.762106 | 513.2742048 |
| LEU-DEG2-PWY | 0.145372163 | 0.010801647 | 3.230894698 | 3.839229376 | 26.27056911 | 41.09272728 |
| LPSSYN-PWY | 0.248799293 | 0.055459904 | 0.355904609 | 1.36824666 | 25.57690998 | 86.14612427 |
| METH-ACETATE-PWY | 0.101150165 | 0.002505267 | 3153.28453 | 1194.920135 | 2271.434041 | 852.8239826 |
| METHANOGENESIS-PWY | 0.180625849 | 0.024605379 | 0.839309818 | 2.513260612 | 100.1015242 | 195.7367343 |
| NAGLIPASYN-PWY | 0.180625849 | 0.017411645 | 1831.90661 | 587.8330709 | 2301.548214 | 712.3073686 |
| NONOXIPENT-PWY | 0.101150165 | 0.002194238 | 11936.70822 | 589.3976363 | 11333.16486 | 737.0382482 |
| P125-PWY | 0.180625849 | 0.020321024 | 166.0502654 | 220.9807271 | 72.11058079 | 71.77550165 |
| P164-PWY | 0.215426045 | 0.045809932 | 3054.496905 | 917.9955093 | 2515.737439 | 760.707962 |
| P241-PWY | 0.180625849 | 0.024605379 | 0.705391857 | 2.063367561 | 80.38355538 | 155.8147242 |
| P261-PWY | 0.215426045 | 0.046019805 | 10.87057269 | 20.15035702 | 87.86757938 | 149.0991083 |
| P42-PWY | 0.203051995 | 0.036461349 | 5318.107737 | 1334.893104 | 6096.30478 | 1229.58451 |
| P562-PWY | 0.129146102 | 0.007196996 | 806.7721719 | 589.9594452 | 488.0088419 | 253.687641 |
| POLYAMINSYN3-PWY | 0.185807077 | 0.028762705 | 561.15883 | 279.4221345 | 760.8111505 | 289.5271122 |
| POLYAMSYN-PWY | 0.140801844 | 0.009590218 | 593.645143 | 267.5313951 | 859.8466681 | 355.2568335 |
| POLYISOPRENSYN-PWY | 0.211247264 | 0.041857043 | 3816.985534 | 710.6495351 | 4247.626924 | 773.7782197 |
| PRPP-PWY | 0.18438162 | 0.027400365 | 2159.647482 | 892.3653654 | 2747.07662 | 804.4579204 |
| PWY-1269 | 0.180625849 | 0.016524947 | 1883.510257 | 566.2875924 | 2387.408865 | 718.2380714 |
| PWY-5028 | 0.211247264 | 0.040682694 | 43.28768192 | 77.31983734 | 17.91944355 | 45.4066084 |
| PWY-5154 | 0.180625849 | 0.02248344 | 2768.442479 | 860.5878191 | 3343.833731 | 909.8075343 |
| PWY-5188 | 0.027486987 | 8.51E-05 | 3577.50267 | 536.2470146 | 2982.521751 | 446.7660199 |
| PWY-5189 | 0.076277699 | 0.001180769 | 3222.882925 | 543.1719733 | 2733.164166 | 429.5158354 |
| PWY-5198 | 0.180625849 | 0.024605379 | 0.481006948 | 1.433166117 | 58.40308128 | 114.9620614 |
| PWY-5304 | 0.121557301 | 0.005345342 | 2878.855386 | 1127.719781 | 2007.038677 | 689.5556803 |
| PWY-5505 | 0.102469149 | 0.002855178 | 7115.692558 | 908.3987404 | 6291.947876 | 893.6153773 |
| PWY-5507 | 0.203051995 | 0.036201276 | 26.55534117 | 30.76120331 | 106.1062565 | 143.735165 |
| PWY-5695 | 0.215426045 | 0.045809932 | 5598.207092 | 882.0006828 | 5974.738945 | 646.7107863 |
| PWY-5741 | 0.140801844 | 0.009081883 | 0.547738396 | 2.08879169 | 19.27181164 | 38.70777838 |
| PWY-6141 | 0.180625849 | 0.024605379 | 0.506545147 | 1.517372 | 60.88290181 | 118.2956625 |
| PWY-6148 | 0.180625849 | 0.024605379 | 1.698641756 | 4.985444227 | 139.9799903 | 259.7707625 |
| PWY-6167 | 0.180625849 | 0.024605379 | 1.595395492 | 4.752821454 | 161.847474 | 301.3737777 |
| PWY-6174 | 0.180625849 | 0.024605379 | 0.94467569 | 2.77709841 | 78.50108004 | 161.4469803 |
| PWY-621 | 0.121557301 | 0.004448978 | 8302.177714 | 1405.79704 | 7327.652269 | 1173.325193 |
| PWY-6263 | 0.180625849 | 0.02233947 | 517.0963632 | 544.7020697 | 789.6748558 | 474.94592 |
| PWY-6317 | 0.221020139 | 0.047899101 | 7771.29214 | 594.4258865 | 7405.24022 | 646.177683 |
| PWY-6349 | 0.180625849 | 0.024605379 | 0.578778808 | 1.733679067 | 67.87207222 | 130.3894731 |
| PWY-6350 | 0.180625849 | 0.024605379 | 0.542666579 | 1.625539609 | 64.40704822 | 124.4427249 |
| PWY-6467 | 0.18438162 | 0.027400365 | 1599.882255 | 514.0354726 | 2008.709303 | 650.3032145 |
| PWY-6519 | 0.215426045 | 0.045809932 | 2100.466998 | 784.3636081 | 2666.966382 | 1082.596845 |
| PWY-6545 | 0.18438162 | 0.027400365 | 1984.604074 | 527.9238663 | 2236.364552 | 413.4145145 |
| PWY-6641 | 0.140801844 | 0.00930398 | 10.52398618 | 20.90719357 | 51.80855105 | 62.34673462 |
| PWY-6654 | 0.180625849 | 0.024605379 | 0.867563453 | 2.598270409 | 100.4522332 | 192.3275464 |
| PWY-6728 | 0.180625849 | 0.020074635 | 1.817509919 | 6.393396661 | 46.86822351 | 85.39388247 |
| PWY-6892 | 0.076277699 | 0.001099568 | 6561.929907 | 733.8247612 | 5860.082478 | 612.4072089 |
| PWY-7003 | 0.192942656 | 0.031659321 | 1249.874664 | 448.2482519 | 991.7292023 | 595.2519929 |
| PWY-7111 | 0.101150165 | 0.002345138 | 11110.31388 | 1216.605891 | 10311.47806 | 897.4171879 |
| PWY-7198 | 0.185807077 | 0.028762705 | 1399.775111 | 315.0734917 | 1199.553267 | 328.5417336 |
| PWY-7199 | 0.121557301 | 0.005030254 | 4198.587246 | 706.9034088 | 4716.154503 | 520.7735317 |
| PWY-7200 | 0.227765661 | 0.050066136 | 2555.577829 | 558.8096116 | 2835.176087 | 475.6166044 |
| PWY-7210 | 0.187475172 | 0.030181762 | 1607.778675 | 328.2997283 | 1382.937702 | 344.3109861 |
| PWY-7234 | 0.145372163 | 0.01072669 | 1019.263483 | 496.0909301 | 1567.073087 | 848.1278208 |
| PWY-7237 | 0.121557301 | 0.006028278 | 4089.137943 | 1994.946301 | 2711.843983 | 1250.395485 |
| PWY-7286 | 0.180625849 | 0.024605379 | 0.578921634 | 1.734171097 | 70.55775317 | 138.2396258 |
| PWY-7315 | 0.140801844 | 0.008560319 | 2138.312444 | 926.2960597 | 1464.761396 | 599.6848171 |
| PWY-7371 | 0.18438162 | 0.025780883 | 248.3138563 | 327.0839425 | 375.6725905 | 267.4341039 |
| PWY-7374 | 0.17233546 | 0.013872204 | 190.2330217 | 304.0105261 | 304.0493902 | 208.9862732 |
| PWY-7391 | 0.194958121 | 0.032793644 | 1.424297521 | 4.041879404 | 40.72448167 | 152.5009086 |
| PWY0-1061 | 0.041405673 | 0.000384573 | 4360.951954 | 949.2956791 | 3467.267274 | 790.1286406 |
| PWY0-1296 | 0.121557301 | 0.005345342 | 6472.025296 | 698.5848668 | 5809.245121 | 976.6816643 |
| PWY0-1297 | 0.205596979 | 0.038191389 | 5481.043211 | 598.0856002 | 5098.982857 | 740.2965121 |
| PWY0-1298 | 0.17233546 | 0.013355625 | 5474.950584 | 492.9306913 | 5022.073492 | 709.5979341 |
| PWY0-1586 | 0.205596979 | 0.038191389 | 6565.992462 | 1466.783752 | 5720.109815 | 1051.635496 |
| superpathway of arginine and polyamine biosynthesis | 0.121557301 | 0.006028278 | 1040.870306 | 425.7085152 | 1453.804719 | 507.8295161 |
| superpathway of fucose and rhamnose degradation | 0.194958121 | 0.033197203 | 1949.739696 | 424.7236232 | 1680.74325 | 342.8398076 |
| THISYN-PWY | 0.200705591 | 0.034797254 | 3688.521716 | 765.7481086 | 4157.031776 | 701.6522787 |
| TRPSYN-PWY | 0.187475172 | 0.030181762 | 5847.830694 | 548.7526986 | 5483.825138 | 441.7803437 |

RRMS: relapsing-remitting multiple sclerosis

HC: healthy controls

SD: Standard Deviation

**Term Descriptions**:

Document: microbiome sample

Term: individual microbe in each sample

Topic: community of microbes

Topic-term probability matrix (beta matrix): the probability a topic (community) is assigned a term (bacteria).

Document-topic probability matrix (gamma matrix): the probability a document (sample) is assigned a topic (community).

Document-term probability matrix: the probability a document (sample) is assigned a term (bacteria). We multiplied the document-topic matrix by the read counts for each sample to assign reads to each topic.

Cosine similarity: a method to compare similarity of two vectors in NLP. Here our vectors were topics (communities) found in either the Chen or Yadav datasets.
